# A computational *in silico* approach to predict high-risk coding and non-coding SNPs of human *PLCG1* gene

**DOI:** 10.1371/journal.pone.0260054

**Published:** 2021-11-18

**Authors:** Safayat Mahmud Khan, Ar-Rafi Md. Faisal, Tasnin Akter Nila, Nabila Nawar Binti, Md. Ismail Hosen, Hossain Uddin Shekhar

**Affiliations:** Department of Biochemistry and Molecular Biology, Clinical Biochemistry and Translational Medicine Laboratory, University of Dhaka, Dhaka, Bangladesh; University of Akron, UNITED STATES

## Abstract

*PLCG1* gene is responsible for many T-cell lymphoma subtypes, including peripheral T-cell lymphoma (PTCL), angioimmunoblastic T-cell lymphoma (AITL), cutaneous T-cell lymphoma (CTCL), adult T-cell leukemia/lymphoma along with other diseases. Missense mutations of this gene have already been found in patients of CTCL and AITL. The non-synonymous single nucleotide polymorphisms (nsSNPs) can alter the protein structure as well as its functions. In this study, probable deleterious and disease-related nsSNPs in *PLCG1* were identified using SIFT, PROVEAN, PolyPhen-2, PhD-SNP, Pmut, and SNPS&GO tools. Further, their effect on protein stability was checked along with conservation and solvent accessibility analysis by I-mutant 2.0, MUpro, Consurf, and Netsurf 2.0 server. Some SNPs were finalized for structural analysis with PyMol and BIOVIA discovery studio visualizer. Out of the 16 nsSNPs which were found to be deleterious, ten nsSNPs had an effect on protein stability, and six mutations (L411P, R355C, G493D, R1158H, A401V and L455F) were predicted to be highly conserved. Among the six highly conserved mutations, four nsSNPs (R355C, A401V, L411P and L455F) were part of the catalytic domain. L411P, L455F and G493D made significant structural change in the protein structure. Two mutations-Y210C and R1158H had post-translational modification. In the 5’ and 3’ untranslated region, three SNPs, rs139043247, rs543804707, and rs62621919 showed possible miRNA target sites and DNA binding sites. This *in silico* analysis has provided a structured dataset of *PLCG1* gene for further *in vivo* researches. With the limitation of computational study, it can still prove to be an asset for the identification and treatment of multiple diseases associated with the target gene.

## Introduction

Single nucleotide polymorphisms (SNPs) are the most common genetic variations found in humans (3–5 million) [1]. It is a type of polymorphism in which a single nucleotide differs between individuals. SNPs of coding region cause the change in amino acid sequences, resulting in an alteration of protein function and hence are termed non-synonymous SNPs (nsSNPs). It has been proven that these mutations show molecular effects with actual phenotypes [1]. Half of the SNPs are nsSNPs and these nsSNPs can affect the protein, both structurally and functionally [2, 3]. Moreover, mutations in the highly structured non-coding regions of the gene can have a significant impact on gene expression. Mutations in the 5’ and 3’ untranslated region can alter the secondary structure of the protein, and thus the binding of proteins and ligands to these regions [4].

Phospholipase C gamma-1 (*PLCG1)* gene has been found associated with noteworthy T-cell lymphomas like peripheral T-cell lymphoma (PTCL), angioimmunoblastic T-cell lymphoma (AITL), cutaneous T-cell lymphoma (CTCL) and adult T-cell leukemia/lymphoma [5–9]. It has also been linked to two subtypes of CTCL- Sezary syndrome and Mycosis fungoides (MF) [10, 11]. Moreover, the mutation of this gene has been found responsible for diseases like bipolar disorder and angiosarcoma [12, 13]. The protein, Phospholipase C gamma-1 (PLCγ1) encoded from the *PLCG1* gene creates inositol 1,4,5-trisphosphate (IP_3_) and diacylglycerol (DAG) from phosphatidylinositol 4,5-bisphosphate (PIP_2_). It is located on chromosome 20 with eight domains. It is bound with calcium while catalyzing the reaction [14]. PLCγ1 also mediates DNA and mRNA synthesis in the process [15]. Epidermal growth factor receptor (EGFR) activates PLCγ1 and helps in cancer cell mitogenesis [16]. It is also suggested that the binding of EGFR-PLCγ1 through SH2 domain is needed for cell cycle progression [16]. An exciting fact is that PLCγ1 can also inhibit cancer cell proliferation by binding with JAK2 and PTP-1B. These two opposite characteristics of the protein make the study of the target gene much more intriguing [14]. The production of DAG and PIP_2_ is in downstream signaling of the T-cell receptor (TCR) pathway, where mutation may cause AITL. S345F and G869E missense mutations have already been found in cases of CTCL and AITL [7]. R707Q missense mutation was found in angiogenesis based lymphoedema angiosarcoma. It is proposed that constitutive angiogenesis signaling driven by PLCγ1 may be the underlying reason for this [13]. K713N missense mutation was found in a sample of MF patient where NF-κB (nuclear factor kappa-light-chain-enhancer of activated B cells), NFAT (Nuclear factor of activated T-cells), and STAT3 (signal transducer and activator of transcription proteins-3) pathways were activated together [11].

No *in silico* analysis of the gene *PLCG1* has been done till now to find all the possible nsSNPs related to the functional and the structural change of the protein. Therefore, the primary purpose of this study was to find possible coding and non-coding SNPs, which can affect the protein function by utilizing various computational approach and bioinformatics tools. These tools find out the possible conserved residues, mutations with the chance of most functionality, possible altered molecular mechanism, structural change in the protein, decreasing protein stability, post-translational modifications (PTM), and other predictable changes to recognize the most significant SNPs [17, 18]. Now-a-days such computational research has become popular to find pathogenicity of genes, such as CSN3, RETN, FOXC2, CHK2 and so on [17–20]. Through our study, it may be possible to identify and predict new SNPs that can be associated with possible diseases.

In this study, we have utilized a number of *in silico* tools to comprehensively characterize the coding and non-coding SNPs located at the *PLCG1* gene. We have shortlisted the most significant nsSNPs and further validated their structural impact through structural analysis. We identified four potentially deleterious nsSNPs (R355C, A401V, L411P and L455F) through our analysis, which form a part of the catalytic domain of PLCG1. Among these, L411P L455F made significant structural changes in the protein structure. Our analysis will provide the framework for further *in vitro* and case-control studies to validate the structural and functional impact of the SNPS in the *PLCG1* gene.

## Materials and methods

### Dataset collection of SNPs

The nsSNP dataset of our target gene *PLCG1* was collected from the dbSNP database (https://www.ncbi.nlm.nih.gov/snp/). After searching for the gene, a missense filter was used to get the nsSNPs. The protein sequence for the gene (FASTA format) was retrieved from the UNIPROT database. To get unique results in our study, SNPs of protein ID ENSP00000362368 were selected. This isoform has been chosen as the canonical sequence. All positional information in this entry refers to it. This is also the sequence that appears in the downloadable versions of the entry [21–23]. For analyzing the non-coding region SNPs, the dataset was collected from ENSEMBL database for the above-mentioned protein ID.

### Detection of deleterious and functional SNPs

Four tools were used to find out the deleterious functional nsSNPs of our dataset. Sorting intolerant from tolerant (SIFT) was adopted in the study to predict whether an amino acid substitution is deleterious or tolerant based on protein conservation with the homology sequence and physical properties of amino acid. Substitution with a probability score of less than 0.05 is considered to be deleterious or functional [24]. Protein variation effect analyzer (PROVEAN) predicts the functional effect of single or multiple amino acid substitutions, insertions, and deletions. The cutoff value for the substitution to be deleterious is -2.5. Anything above is counted as neutral or non-deleterious [25]. Polymorphism Phenotyping version 2 (PolyPhen-2) is a similar kind of tool which predicts damaging missense mutations using multiple sequence alignment and structural information [26]. Protein analysis through evolutionary relationship (PANTHER) does an evolutionary analysis of coding SNPs to find the damaging amino acid substitutions [27].

### Disease related SNPs

The common nsSNPs found to be deleterious in all four previous tools, were then run in 3 disease-associated SNPs predicting tools. Predictor of human Deleterious Single Nucleotide Polymorphisms (PhD-SNP) uses support vector machine (SVM) method to predict whether a phenotype of nsSNP can be related to any disease associated conditions. The output of the result comes with a reliability index predicting if the SNP is disease-causing or neutral [28]. Pathogenic mutation prediction (Pmut) server uses a neural network-based predictor which is trained by a manual database SwissVar to predict if a mutation is associated with a disease or not. A prediction scoring from 0.5–1 is termed as disease-causing [29]. *SNPS&GO* is another SVM based tool which predicts a mutation to be disease-causing based on the protein sequence as well as the protein structure (when available) and gene ontology terms [30, 31].

### Prediction of change in protein stability

The common SNPs found to be disease-causing were then run to check protein stability. The deleterious nsSNPs with decreasing protein stability are considered as substantial. I-mutant 2.0 and MUpro were used to predict the change in protein stability due to the mutations. I mutant 2.0 is another SVM based tool that provides the free energy change (Delta Delta G) value and predicts the sign as increase or decrease. A Delta Delta G (DDG) (kcal/mole) value <0 means a decrease in the protein stability, whereas DDG (kcal/mole) value >0 means an increase in the protein stability [32]. MUpro uses two methods: SVM and neural networks. However, SVM method result is recommended. A confidence score <0 indicates a decrease in protein stability, and a confidence score >0 indicates an increase in protein stability with the mutation [33].

### Prediction of the molecular mechanism of pathogenicity

The common SNPs found to be disease-related from PhD-SNP, Pmut and *SNPS&GO* were run in Mutpred2 server. It is a server that can predict the pathogenicity of the substitutions with a detailed molecular target and affected mechanisms. It uses multiple neural networks, and the final score is the cumulative results from all of them, ranging from 0 to 1. The closer the result is to 1, the higher the chance of the substitution to alter its stability. The threshold of *p* value was set at 0.05; only substitutions with *p* value equal or less than this were collected [34].

### Prediction of post-translational modification

ModPred server was used to see if there were any post-translational modification (PTM) sites in our common target SNPs, which were found to be disease-causing. This server uses a collection of datasets containing 278,703 PTM sites. The tool then assesses those PTM sites for multiple protein sequences. The output gives potential PTM sites for each residue with a confidence score. Only high and medium confidence score PTMs were taken into consideration [35].

### Sequence conservation and solvent accessibility

Again, the disease-causing SNPs’ conservation and solvent accessibility were checked. Consurf predicts the evolutionary conservation of residues of a protein sequence. It estimates the evolutionary rate of the amino acids and further can anticipate if the substitution has any structural or functional effect along with a conservation score ranging from 1–9. Here score 9 indicates the most conserved amino acid, whereas scoring 1 to the most variable. It also provides solvent accessibility of the amino acid residues where the results show if the amino acids are buried or exposed. Generally, they evaluate their result from protein structure, but as our structure was not available on PDB, they predicted the result through protein sequence (Conseq) [36].

Netsurf 2.0 was also used to predict the solvent accessibility of the amino acid residues. It uses a neural network that has been used on protein structures and shows the buried and exposed regions of the protein [37].

### Mutation cluster prediction

Mutation3D is a web-based tool to identify clusters of amino acids which can arise from somatic mutation. It can predict driver genes for mutation, separating the functional SNPs from the nonfunctional ones. The common SNPs found to be disease-related from PhD-SNP, Pmut, and *SNPS&GO* were put along with query sequence to identify possible clusters [38].

### Structural analysis

#### Homology modeling by SWISS-MODEL

The target protein structure was not available on PDB, so the homology modeling of the protein was done by the SWISS-MODEL server. This server takes a query sequence as input, searches for closely related sequence template for the structure and aligns them [39]. Using that structure as template homology modeling was done, and the model was further validated by QMEAN value. It also provides Ramachandran plot to ensure the quality of the generated structure further. The template with maximum coverage and highest sequence identity was chosen. The native wild type protein structure and mutant protein residues’ structures were generated. The mutant residues which had high conservation scores were generated for homology modeling.

#### Model validation

All the structures generated by homology modeling were validated by the tool PROCHECK. It is a standard tool to verify the stereochemical quality of a protein structure. It generates a Ramachandran plot to validate the structure with details of residues in the core and other allowed regions [40].

#### RMSD value and TM align value

The RMSD value associated with the mutant residues after superimposition with the native protein structure was calculated with PyMol, an open-source software to perform structural analysis [41].

TM align value is checked to see the structural dissimilarity between the native and wild type structure. A score of 1 means that there is no dissimilarity between the structures; a score < 0.2 means unrelated protein structures, whereas a score > 0.5 means the same fold [42].

#### Chemical property analysis by BIOVIA discovery studio visualizer

Further analysis of the mutant residue structures compared with the wild type structure was done by BIOVIA discovery studio visualizer, a structural analysis tool [43]. It is downloadable from the website (https://discover.3ds.com/discovery-studio-visualizer-download). It can help to visualize protein structures, residue solvent accessibility, polar and non-polar bonds, and analyze the difference between native and wild type residues. Specific SNPs were selected with a cumulative result of conservation score, solvent accessibility, structural/functional prediction by Consurf, RMSD value, TM align value, Mutation cluster prediction, respectively, and taken into further consideration.

### Analysis of 5’ and 3’ UTR non-coding SNPs

Investigation of non-coding regions was done using the ENSEMBL database [44]. The 5’ and 3’ region SNPs were filtered out. Minor allelic frequency (MAF) value of ≤ 0.001 was selected only. Later the SNPs were run in Regulome DB, which relates SNPs to regulatory elements of the human genome [45]. It gives a ranking based on DNA binding, and also provides Chip data, chromatin states, and motifs, if available. Information of our gene was checked in the PolymiRTS database- a server to predict naturally occurring DNA variation in miRNA target sites, mainly in the 3’ UTR region [46]. The results are given in 4 classes D, O, C and N with a context and conservation score along with miR ID and miR target site.

### Gene-gene interaction analysis

Gene MANIA server was used to correlate the target *PLCG1* gene with functionally similar genes and further analyze the interactions among them [47]. Currently, it supports six organisms with datasets collected from GEO, BioGRID, Pathway commons, I2D etc. Ensemble was used as the primary identifier. Interaction data available between the genes include physical interaction, co-expression, co-localization, genetic interaction.

**Fig 1:**
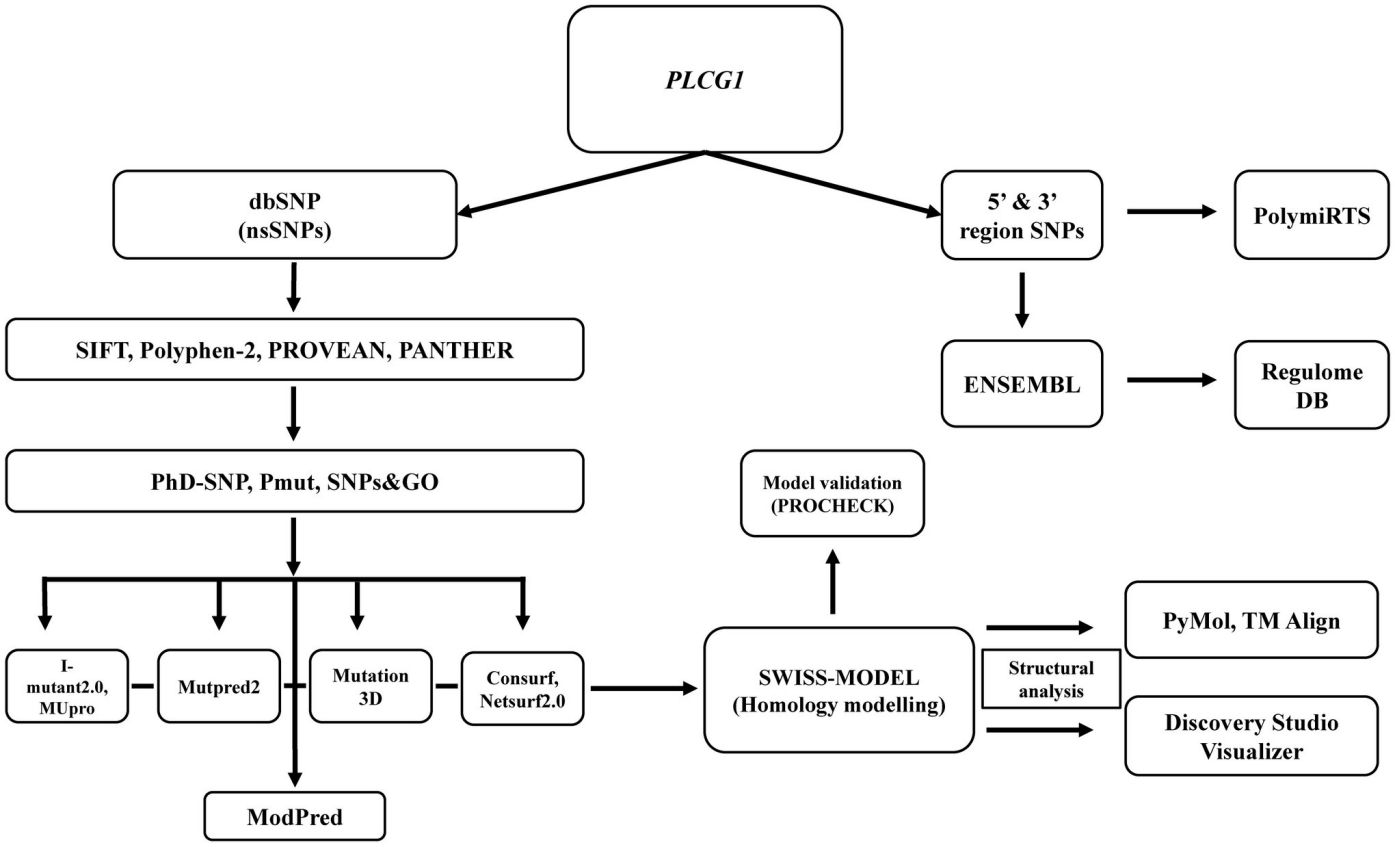
An outline of the methodology used in this study.

An outline of the methodology used in this study has been summarized (Fig 1).

## Results

### SNP datasets

SNPs of the *PLCG1* gene were retrieved from the dbSNP database. Primarily, 11096 SNPs were found, but after applying the missense filter, 745 SNPs were retrieved (https://www.ncbi.nlm.nih.gov/snp/?term=*PLCG1*). Later, protein isoform P19174-1 was selected for the current study, and its sequence was retrieved from the UniProt database to perform the analyses.

### Detection of deleterious and functional SNPs

**Fig 2:**
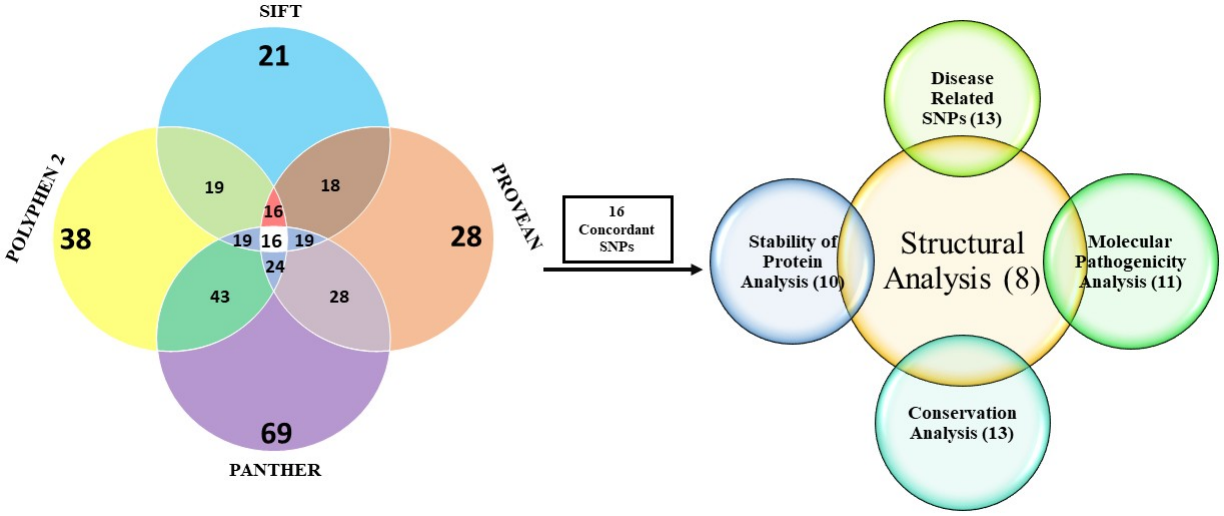
Venn diagram representation of most deleterious nsSNPs estimated by various *in silico* tools. A total of 16 SNPs were showed concordant results as deleterious nsSNPs by SIFT, PolyPhen 2.0, PROVEAN and PANTHER. Further analysis of these SNPs using different in silico tools resulted in 8 nsSNPs, which were selected for structural analysis.

After running the 745 SNPs in the SIFT tool, the result was filtered with our target protein ID and 74 SNPs were obtained. The 74 SNPs were then run in PolyPhen-2, PROVEAN, and PANTHER tools. After combining the results, 16 SNPs were found to be deleterious in all the tools (Table 1). SNPs having neutral or non-deleterious results in any of the tools were not selected for further analyses (Fig 2).

**Table 1:** Prediction of functionality of nsSNPs with SIFT, PROVEAN, Polyphen-2 & PANTHER.

SNP	Amino acid Change	SIFT Prediction	SIFT score	PROVEAN	PROVEAN score	Polyphen-2	Probability Score	PANTHER
**rs373972267**	L411P	Deleterious	0.002	Deleterious	-6.863	probably damaging	1	probably damaging
**rs367808225**	I109T	Deleterious	0.002	Deleterious	-4.06	probably damaging	0.971	probably damaging
**rs202246756**	A816P	Deleterious	0.005	Deleterious	-4.497	probably damaging	1	probably damaging
**rs201158224**	R355C	Deleterious	0.02	Deleterious	-7.485	probably damaging	1	probably damaging
**rs200946488**	R601Q	Deleterious	0.032	Deleterious	-3.413	probably damaging	1	probably damaging
**rs199826230**	Y210C	Deleterious	0.003	Deleterious	-5.022	probably damaging	0.991	possibly damaging
**rs199669312**	P244L	Deleterious	0.036	Deleterious	-3.398	possibly damaging	0.835	possibly damaging
**rs191463364**	G493D	Deleterious	0.037	Deleterious	-6.517	probably damaging	0.964	probably damaging
**rs186053167**	R1105L	Deleterious	0.004	Deleterious	-6.414	probably damaging	0.995	probably damaging
**rs148020473**	P1152A	Deleterious	0.036	Deleterious	-6.908	probably damaging	0.986	probably damaging
**rs147844565**	D1075V	Deleterious	0.031	Deleterious	-7.077	possibly damaging	0.919	probably damaging
**rs147137389**	S345C	Deleterious	0.007	Deleterious	-3.82	probably damaging	1	probably damaging
**rs141684852**	R1158H	Deleterious	0	Deleterious	-4.72	probably damaging	1	probably damaging
**rs7266677**	A401V	Deleterious	0.002	Deleterious	-3.883	probably damaging	1	probably damaging
**rs6065316**	L455F	Deleterious	0.005	Deleterious	-3.92	probably damaging	1	probably damaging
**rs2235361**	I949T	Deleterious	0.002	Deleterious	-3.957	probably damaging	0.999	probably damaging

### Disease related SNPs

The 16 deleterious SNPs were then run in three tools: PhD-SNP, Pmut and *SNPS&GO* to predict if the mutations can be related with diseases or disease associated conditions. Out of the 16 mutations, 13 SNPs showed disease-causing effects in all the three tools (Table 2). Again, any SNP having neutral result in any of the tools mentioned above was not selected for further analyses.

**Table 2:** Prediction of disease associated nsSNPs by Pmut, PhD-SNP & SNPS&GO.

SNP	Amino acid change	Pmut	Prediction score	PhD-SNP	Reliability Index	SNPS & Go	Reliability Index	Probability
**rs373972267**	L411P	Disease	0.927 (94%)	Disease	9	Disease	6	0.816
**rs367808225**	I109T	Disease	0.876(92%)	Disease	8	Disease	2	0.622
**rs202246756**	A816P	Disease	0.725 (87%)	Disease	4	Disease	2	0.584
**rs201158224**	R355C	Disease	0.865 (91%)	Disease	7	Disease	6	0.8
**rs200946488**	R601Q	Disease	0.674 (85%)	Disease	6	Disease	3	0.664
**rs199826230**	Y210C	Disease	0.580 (82%)	Disease	7	Disease	6	0.797
**rs191463364**	G493D	Disease	0.522 (79%)	Disease	6	Disease	0	0.502
**rs186053167**	R1105L	Disease	0.666 (85%)	Disease	4	Disease	7	0.842
**rs148020473**	P1152A	Disease	0.790 (89%)	Disease	6	Disease	2	0.615
**rs147844565**	D1075V	Disease	0.756 (88%)	Disease	5	Disease	4	0.676
**rs141684852**	R1158H	Disease	0.834 (90%)	Disease	9	Disease	5	0.745
**rs7266677**	A401V	Disease	0.866 (91%)	Disease	8	Disease	6	0.817
**rs6065316**	L455F	Disease	0.852 (91%)	Disease	8	Disease	2	0.583

### Prediction of change in protein stability

The 13 disease-associated SNPs were put in I-mutant 2.0 and MuPro to check their effect on protein stability. All the SNPs showed decreasing protein stability in MuPro server, but three SNPs Y210C, A401V and L455F showed increasing stability in the I-mutant 2.0 server (Table 3).

**Table 3:** Prediction of protein stability of nsSNPs by I-mutant 2.0 & MuPro.

SNP	Amino acid change	I-mutant 2.0	DDG value prediction (Kcal/mol)	MuPro	Value (SVM)
**rs373972267**	L411P	Decrease	-0.48	Decrease	-1.642
**rs367808225**	I109T	Decrease	-3.75	Decrease	-2.151
**rs202246756**	A816P	Decrease	-2.75	Decrease	-1.004
**rs201158224**	R355C	Decrease	-0.39	Decrease	-0.614
**rs200946488**	R601Q	Decrease	-1.7	Decrease	-1.125
**rs199826230**	Y210C	Increase	0.91	Increase	0.908
**rs191463364**	G493D	Decrease	-1.58	Decrease	-0.936
**rs186053167**	R1105L	Decrease	-0.71	Decrease	-0.548
**rs148020473**	P1152A	Decrease	-1.83	Decrease	-1.379
**rs147844565**	D1075V	Decrease	-1.04	Decrease	-0.738
**rs141684852**	R1158H	Decrease	-2.64	Decrease	-1.759
**rs7266677**	A401V	Increase	0.07	Decrease	-1.759
**rs6065316**	L455F	Increase	0.47	Decrease	-0.871

### Prediction of the molecular mechanism of pathogenicity

The 13 common SNPs were run in MutPred2 server to check protein stability alteration capability and molecular effect of the mutations. Out of them, 11 SNPs showed a satisfactory result within the threshold range. The functional impacts included altered stability, loss of DNA strand, altered metal binding, gain of helix, loss of phosphorylation sites, altered ordered interface, and gain of relative solvent accessibility. Details of the result along with *p* value and prediction score are given (Table 4).

**Table 4:** Effect of nsSNPs on the structure and function of protein predicted by Mutpred2.

SNP	Amino acid change	MutPred2 score	Effect	P value
**rs373972267**	L411P	**0.934**	Altered Metal binding	0.02
			Altered stability	0.01
**rs367808225**	I109T	**0.815**	Altered Metal binding	0.04
			Altered stability	0.01
**rs202246756**	A816P	**0.907**	Altered Ordered interface	0.02
			Gain of Loop	0.04
			Altered Transmembrane protein	1.50E-03
			Gain of Relative solvent accessibility	0.04
**rs201158224**	R355C	**0.859**	Altered Ordered interface	8.30E-03
**rs200946488**	R601Q	**0.725**	Loss of Strand	0.02
			Altered Ordered interface	0.05
			Altered DNA binding	0.01
**rs199826230**	Y210C	**0.577**	Loss of Phosphorylation at Y210	0.02
**rs191463364**	G493D	**0.841**	Altered Transmembrane protein	4.80E-04
			Gain of Helix	0.05
			Loss of Strand	0.03
**rs148020473**	P1152A	**0.618**	Altered Transmembrane protein	0.03
**rs141684852**	R1158H	**0.796**	Altered Ordered interface	0.04
			Loss of Strand	0.04
			Altered Transmembrane protein	1.60E-03
			Altered Metal binding	0.01
			Gain of Sulfation at Y1162	1.30E-03
			Altered Stability	0.04
**rs7266677**	A401V	**0.859**	Altered Metal binding	4.60E-03
**rs6065316**	L455F	**0.799**	Loss of Relative solvent accessibility	0.02
			Gain of Strand	0.03
			Gain of Acetylation at K456	0.04

### Prediction of post-translational modification

ModPred server provided significant results for two of the SNPs: Y210C and R1158H. Both the SNPs had post-translational modification in the native and mutant residue. Y210C showed proteolytic cleavage in the native residue and amidation modification in the mutant residue. R1158H had proteolytic cleavage in both mutant and native residues (Table 5).

**Table 5:** Prediction of post-translational modification site of SNPs by ModPred.

SNP	Amino acid change	Native residue	Modification Type	Score	Confidence level	Mutant residue	Modification type	Score	Confidence level
**rs199826230**	Y210C	Tyrosine	Proteolytic cleavage	0.77	Medium	Cysteine	Amidation	0.75	Medium
**rs141684852**	R1158H	Arginine	Proteolytic cleavage	0.9	High	Histidine	Proteolytic cleavage	0.86	Medium

### Sequence conservation and solvent accessibility

All the 13 SNPs had good conservation scores in Consurf, but only the ones with score 8 and 9, and prediction of effect in MutPred2 server were chosen for further structural analysis. Six SNPs (L411P, R355C, G493D, R1158H, A401V and L455F) scored 9. Among them, L411P, G493D, A401V and L455F were shown to have possible structural effects as they were highly conserved and buried. The rest two SNPs were shown to have a possible functional effect as they were highly conserved exposed residues. Netsurf 2.0 showed contradictory results in three SNPs. R355C and R1158H were shown as buried residues instead of exposed shown by Consurf. In Netsurf 2.0, Y210C was shown as exposed residue, unlike Consurf where it was shown as buried. The details are given (Table 6), and the conservation score prediction figure is given (S1 Fig).

**Table 6:** Conservation prediction & solvent accessibility analysis of selected nsSNPs by Consurf & Netsurf 2.0.

SNP	Amino acid change	Consurf conservation score	Buried/Exposed (Consurf)	Buried/Exposed (Netsurf 2.0)	Disorder probability (Netsurf 2.0)
**rs373972267**	L411P	9	B	b	9.64E-05
**rs367808225**	I109T	7	B	b	8.71E-06
**rs202246756**	A816P	8	B	b	0.001336
**rs201158224**	R355C	9	E	b	0.000234
**rs200946488**	R601Q	8	E	e	0.001724
**rs199826230**	Y210C	7	B	e	0.002218
**rs191463364**	G493D	9	B	b	0.010974
**rs186053167**	R1105L	8	E	e	0.045405
**rs148020473**	P1152A	7	E	e	0.000897
**rs147844565**	D1075V	6	E	e	0.005478
**rs141684852**	R1158H	9	E	b	3.82E-05
**rs7266677**	A401V	9	B	b	0.00229
**rs6065316**	L455F	9	B	b	0.003929

b: Buried; e: Exposed.

### Mutation cluster prediction

The 13 disease-causing SNPs were used to predict the mutation clusters. Mutation 3D showed a cluster in the PI-PLC-X domain consisting of three substitutions L411P, L455F, and A401V. There can be other clusters but not shown in the prediction tool because of the unavailability of the whole structure in the tool’s database. While doing structural analysis, this criterion was taken into account. The result is given along with other structural information (Table 7).

**Table 7:** Structural analysis of highly conserved residues by various tools.

SNP	Amino acid change	TM align value	RMSD value	Residues in core region (Procheck)	Total Hydrogen Bonds (BIOVIA Discovery Studio visualizer)	Mutation cluster
**rs373972267**	L411P	0.98036	2.423	88.4%	1310	Cluster
**rs141684852**	R1158H	0.99	0.062	87.9%	1260	-
**rs7266677**	A401V	1.0	0.011	88.3%	1292	Cluster
**rs6065316**	L455F	0.99998	0.096	88.2%	1278	Cluster
**rs201158224**	R355C	0.99988	0.195	88.2%	1276	-
**rs191463364**	G493D	0.98644	1.973	88.4%	1329	-
**rs202246756**	A816P	0.99998	0.092	88%	1274	-
**rs200946488**	R601Q	1.0	0.044	88.3%	1285	-

*Native protein structure has 1293 hydrogen bonds;

“-” means no cluster.

### Structural analysis

#### Homology modeling

**Fig 3:**
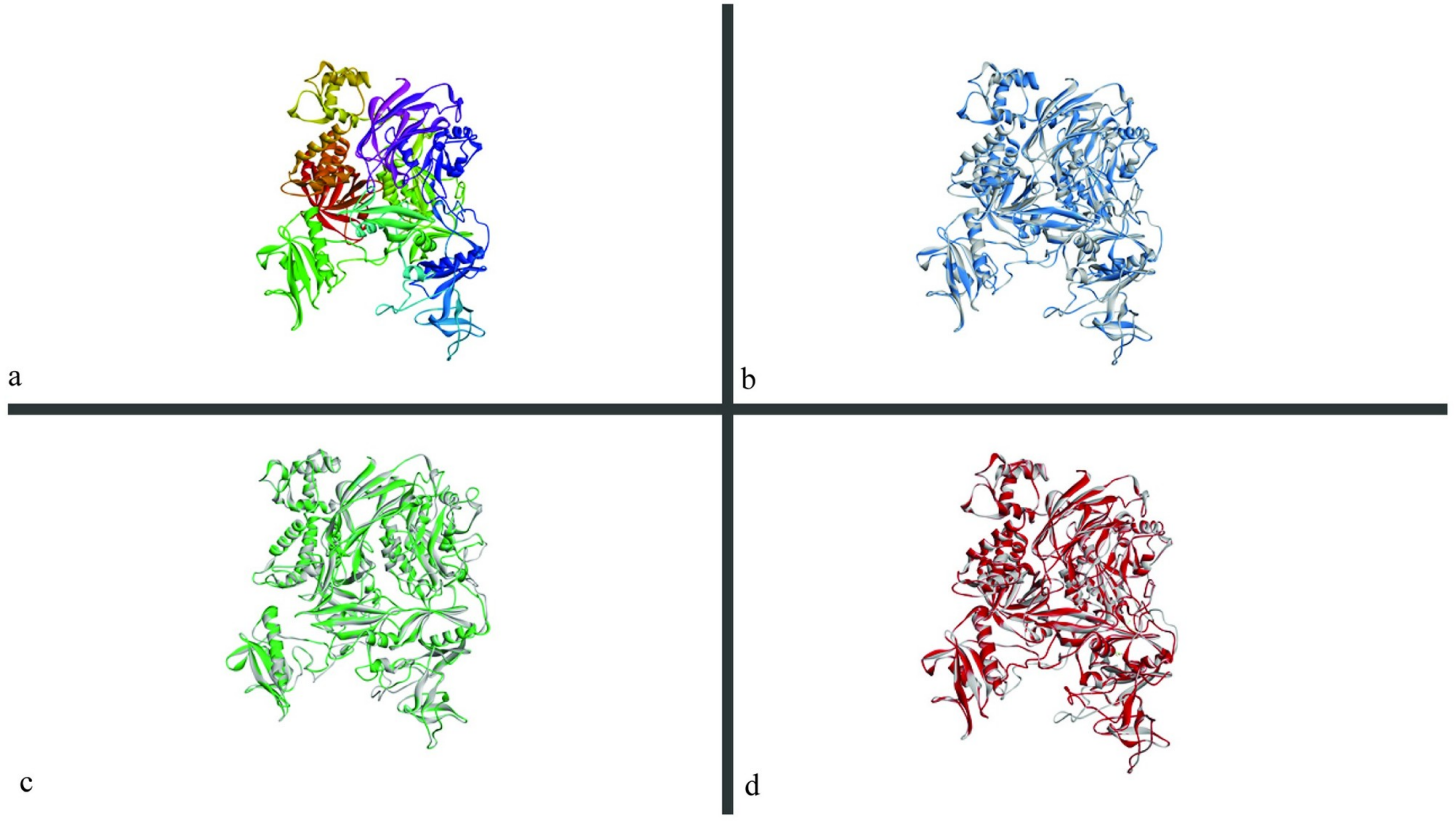
(a) Native wild type structure made by SWISS-MODEL. (b) Superimposed image of native protein structure onto mutant L455F (blue) protein structure, (c) mutant L411P (green) protein structure, (d) mutant G493D (red) protein structure. (Visualized by BIOVIA discovery studio visualizer).

Eight SNPs (L411P, R355C, G493D, R1158H, A401V, L455F, A816P and R601Q) were chosen for structural analysis (Fig 2). 6pbc.1. A template (X-ray structure) was used to generate our native and mutant protein structures in the SWISS-MODEL server. It had 97.19% sequence identity and 91% coverage. All the targeted SNPs were in the covered region. The native structure of the protein is shown (Fig 3).

#### Model validation

All the structures generated from SWISS-MODEL were given in the PROCHECK tool. It showed almost 90% residues in the core region for all the structures. The results of core region residues are given (Table 7), and the quality assessment of structure and data are given (S3–S11 Figs).

#### RMSD value and TM align value

The RMSD values of the eight SNPs were calculated by PyMol software. Among them, five SNPs (L411P, L455F, R355C, G493D and A816P) showed a high deviation. Their TM-align value was also checked, and all five SNPs showed fluctuation in their property. The results are given (Table 7).

#### Chemical property analysis by BIOVIA discovery studio visualizer

**Fig 4:**
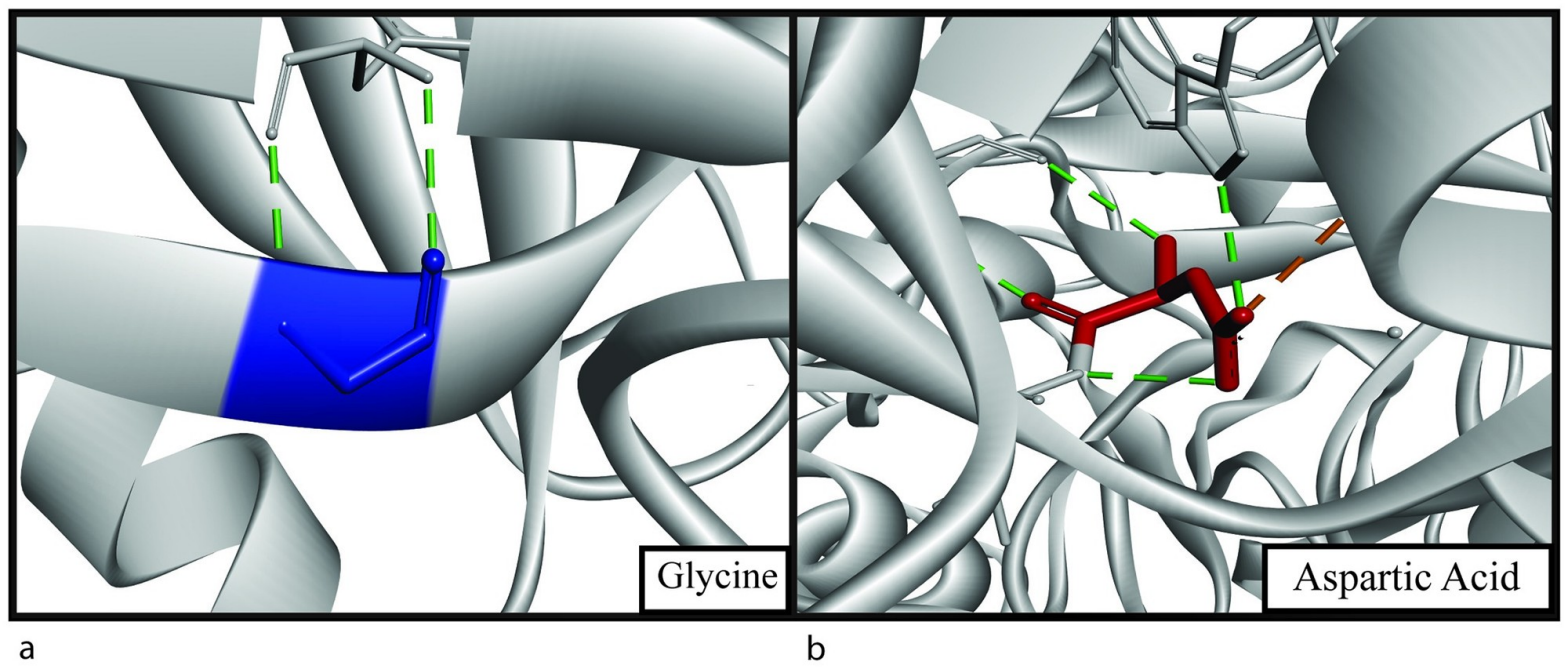
(a) Structural analysis showing Gly493 (blue) of native structure having 2 hydrogen bonds (green) and (b) Asp493 (red) of mutant structure having 4 hydrogen bonds (green) and a salt-bridge bond (orange).

**Fig 5:**
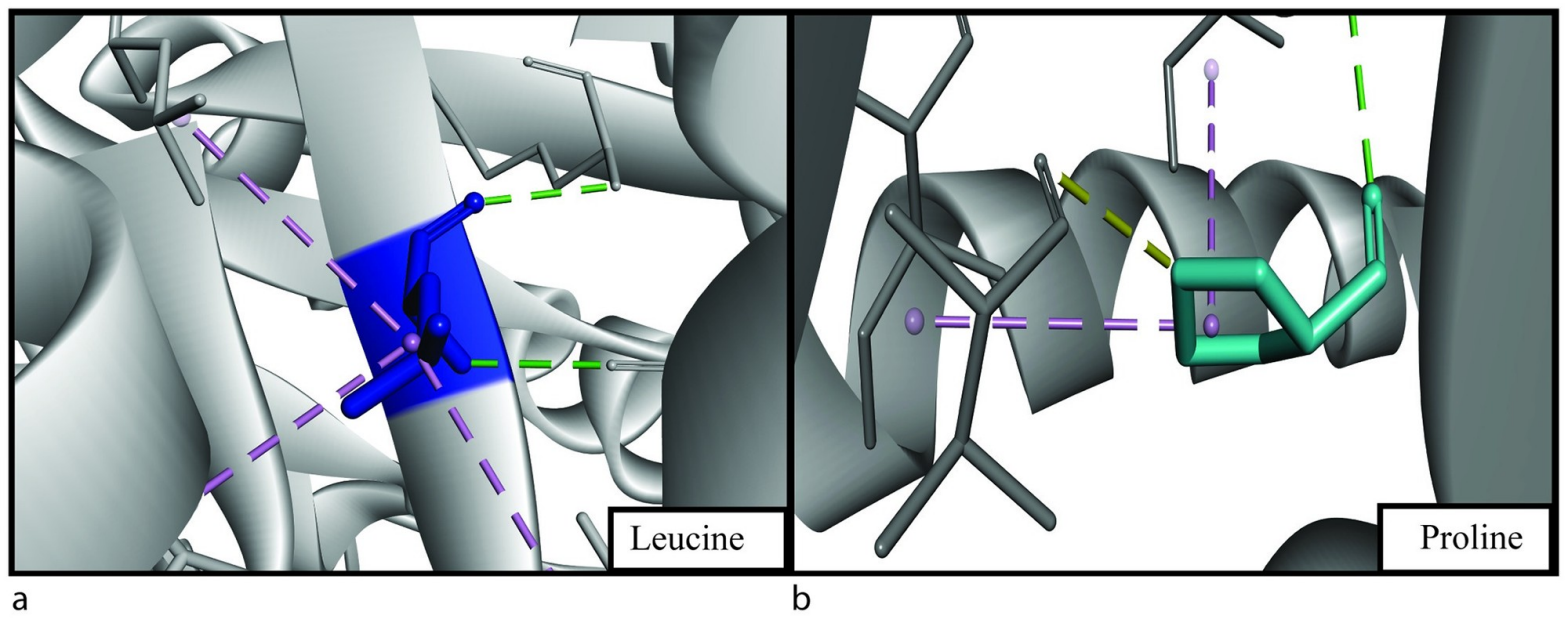
(a) Structural analysis showing Leu411 (blue) of native structure having 2 hydrogen bonds (green), 3 hydrophobic alkyl bonds (purple) and (b) Pro411 (turquoise) of mutant structure having a hydrogen bond (green), a carbon-hydrogen bond (yellow) and 2 alkyl hydrophobic bonds (purple).

**Fig 6:**
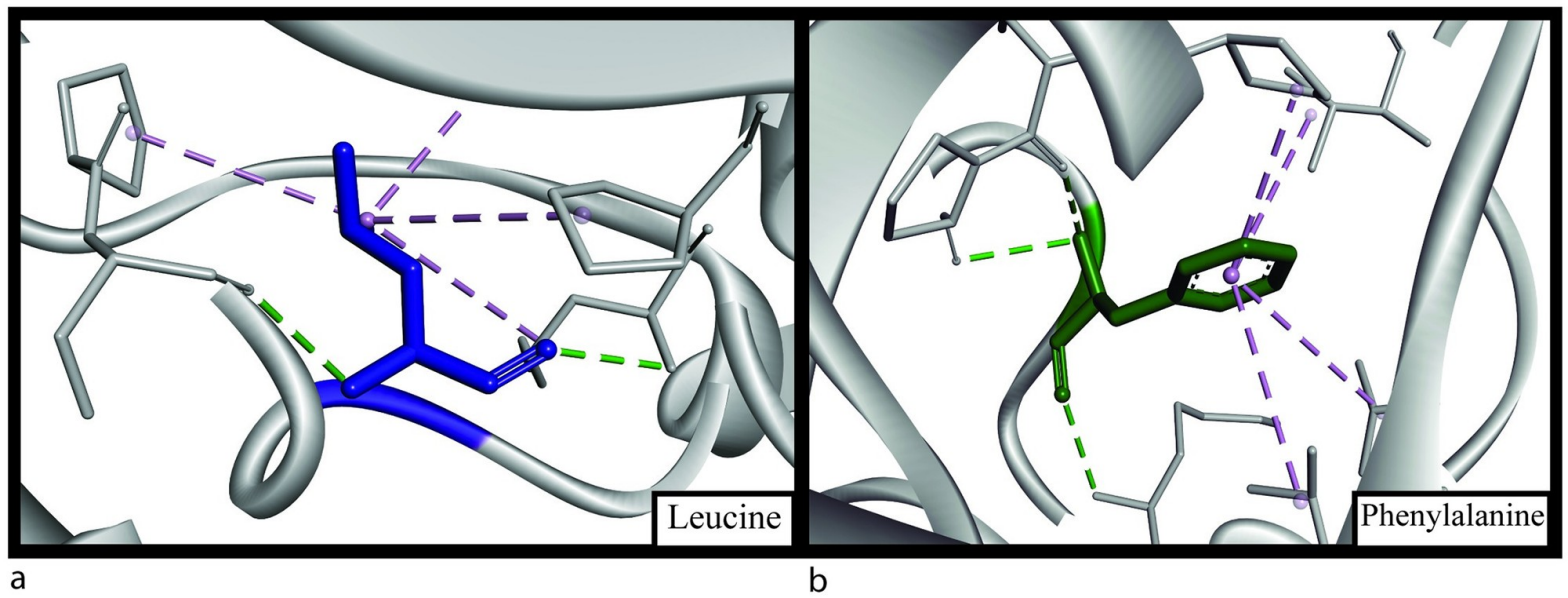
(a) Structural analysis showing Leu455 (blue) of native structure having 2 hydrogen bonds (green), 4 hydrophobic alkyl bonds (purple) and (b) Phe455 (green) of mutant structure having 3 hydrogen bonds (green) and 4 hydrophobic alkyl bonds (purple).

**Fig 7:**
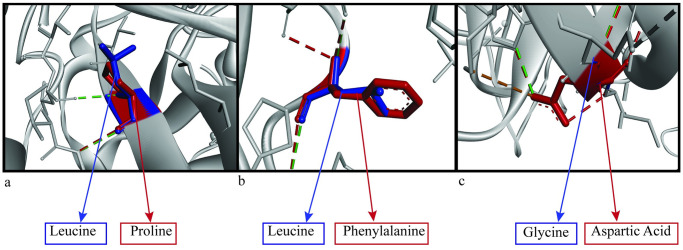
Superimposed protein structures of native and mutant structures (a) L411P, (b) L455F and (c) G493D showing comparison of hydrogen bonds. Blue color shows native residues, Green color shows hydrogen bonds of native residues and Red color shows mutant residues and their hydrogen bonds.

A filtration of SNPs was done for further analysis of our protein structures. Total hydrogen bonds of all the eight SNPs were generated by the BIOVIA discovery studio visualizer (Table 7). Then taking account of RMSD value, TM align value, total hydrogen bonds, and mutation cluster prediction, three SNPs (G493D, L411P and L455F) were chosen for further chemical analysis. The three SNPs had RMSD values of 2.423Å, 0.096Å, and 1.973Å, respectively. They had TM align value showing differences in the structures and the hydrogen bonds compared to the wild structures. L411P and L455F showed mutation cluster in the prediction by Mutation 3D (Table 7). Three SNPs showed changes in hydrophobicity and number of hydrogen bonds, after further analysis by BIOVIA discovery studio visualizer (Table 8). The mutant protein structures of the three SNPs are given (Fig 3b–3d). The intermolecular bonds generated by the wild type and mutant structures of the three SNPs-G493D, L411P and L455F, are shown respectively (Figs 4–6). Finally, the comparative superimposed structures showing hydrogen bonds and their difference in numbers and angles are shown (Fig 7).

**Table 8:** Chemical analysis result of target SNPs by BIOVIA discovery studio visualizer.

SNP	Amino acid Position		Residue	Hydrophobicity	Secondary structure	Number of Hydrogen Bonds (Range)
**rs191463364**	493	Native	Glycine	-3.5	Sheet	2 (493G-510F, 510F-493G)
		Mutant	Aspartic acid	-0.4	Sheet	4 (493D-510F, 510F-493D, 494I-493D, 922W-493D)
**rs373972267**	411	Native	Leucine	3.8	Sheet	2 (411L-460L, 462K-411L)
		Mutant	Proline	-1.6	Sheet	1 (462K-411P)
**rs6065316**	455	Native	Leucine	3.8	Turn	2 (455L-451S, 458K-455L)
		Mutant	Phenylalanine	2.8	Turn	3 (455F-451S, 455F-452P, 458K-455F)

### Analysis of 5’ and 3’ UTR non-coding SNPs

After setting the MAF filter of ≤0.001, 65 SNPs were found in the Ensemble database. In Regulome DB only the SNPs with ranking < 4 were taken into consideration, and nine SNPs were chosen. The rankings along with probability score and Chip data are given (Table 9). In the S1 section, data of all the SNPs of Regulome DB generated from ENSEMBLE has been given (S7 Table).

**Table 9:** Regulome DB data for non-coding SNPs of PLCG1.

SNP	Probability score	Ranking	Chip DATA
**rs139043247**	0.6	2a	POLR2A, ESR1, ZIC2
**rs543804707**	0.604	2b	POLR2A, ESR1, ZIC2
**rs532229042**	0.29248	3a	POLR2A, RBFOX2, NRF1, SIN3A, YY1, POLR2G, ZNF592, DPF2, PHF8, AGO2
**rs571170027**	0.30476	3a	POLR2A, PAF1
**rs535979515**	0.81114	3a	POLR2A, PAF1
**rs62621919**	0.72923	3a	POL2RA
**rs182769107**	0.6352	3a	POL2RA
**rs114288140**	0.66203	3a	POLR2A, PAF1
**rs551768008**	0.90505	3a	POLR2A

2a: TF binding + matched TF motif + matched DNase Footprint + DNase peak; 2b: TF binding + any motif + DNase Footprint + DNase peak; 3a: TF binding + any motif + DNase peak.

PolymiRTS database provided data with miRNA target sites for *PLCG1* gene. Among the SNPs which provided results in Regulome DB, two SNPs rs139043247 and rs62621919 also provided result in the PolymiRTS database. rs139043247 has two alleles G and A in the database with class of D and C, respectively in all their target sites. All the target sites came with negative context scores and a high conservation score. rs62621919 has two alleles G and A with class of D and C, respectively in their target sites along with negative context scores (S4–S6 Tables).

### Gene-gene interaction analysis

GENEMANIA interaction analysis showed strong interaction of 20 genes, including oncogenes like *KIT*, *FYN*, *RET*, *CBL* with *PLCG1*. Immunity-related genes like *ITK*, *EPOR*, *PECAM1* interact with *PLCG1*. The figure of the interaction of *PLCG1* with all possible genes is given (S2 Fig).

## Discussion

The target gene, human *PLCG1* produces the protein PLCγ1, which consists of an N-terminal PH domain followed by EF hands, TIM barrel (X and Y), and a C-terminal domain C2. There is an insertion of two parts of another PH domain between the TIM barrel catalytic domain. The two parts of PH2 domain are further split into two SH domains and one SH3 domain [48]. It is a monomer with two isoforms found in the human body (P19174-1, P19174-2). Our selected isoform P19174-1 had 1290 amino acid residues in it, and all the SNPs predicted to be damaging or having mutations with functional effects are scattered in these domains. The prediction of nsSNPs has been very significant in recent years as these mutations have been related to several diseases, and computational approach has become a successful way to predict them quite efficiently [17–20]. As no *in silico* analysis has been done to date to predict deleterious nsSNPs and possible functional non-coding SNPs associated with our target gene *PLCG1*, the purpose of this analysis was to find out possible nsSNPs and non-coding SNPs which can affect the functionality of the protein molecule.

Several tools were used to predict the probable damaging effect of nsSNPs of *PLCG1* gene. At first, the nsSNPs gathered from the dbSNP database were filtered out according to the prediction of their functionality. Using four tools SIFT, PROVEAN, PolyPhen-2 and PANTHER, 16 SNPs were considered to have deleterious effects. These tools generally use the idea of finding the more conserved residues to predict the effect. Similar tools that predict mutations associated with diseases are PhD-SNP, Pmut and SNPS&Go. These tools filtered out three SNPs, and thus, 13 SNPs were finalized for further study. From the Uniprot database, the following information was obtained: **I109T** substitution is from Domain **PH1** (27–142); **R355C, A401V, L411P and L455F** substitutions are from **PI-PLC X box** domain (320–464); **G493D** substitution is from first part of **PH2** domain (489–523); **R601Q** substitution is from one of the **SH2** domains (550–657); **A816P** substitution is from **SH3** domain (791–851) “**R1105L, P1152A, D1075V** and **R1158H”** substitutions are from the C terminal **C2** domain (1071–1194). These are important details as different domains are associated with different activities, and nsSNPs of these domains may alter their structures and activities. The four nsSNPs of the X box domain are the most significant ones as this domain is part of catalytic activity [48]. On the other hand, the C2 domain is involved in calcium-binding of the protein and subcellular localization, so nsSNPs of this domain can be considered important also [49]. SH2 domain is crucial for cancer cell cycle progression, [16] as a result, R601Q can be a significant nsSNP. From the cBioPortal database, it was found out that G493D and R355C mutations were found in patient samples [50]. The G493D mutation was found in Uterine mixed endometrial carcinoma patient sample, and the R355C mutation was found in the Leiomyosarcoma patient sample. The link of the result is given. (https://www.cbioportal.org/results/mutations?cancer_study_list=5c8a7d55e4b046111fee2296&case_set_id=all&gene_list=PLCG1).

After finalizing the 13 SNPs, the effect of these SNPs on protein stability was checked. Decreasing protein stability with the effect of substitution indicates the possible effect of SNPs on proteins [32]. The Gibbs free energy is directly related to protein stability. A value <0 indicates decreasing protein stability [51]. Almost all the proteins showed decreasing result for both the tools- I-mutant 2.0 and MuPro. ModPred predicted PTM sites in Y210C and R1158H for native and mutant residues. Y210C had proteolytic cleavage for its native residue, which is a very important modification as it can produce irreversible post-translational modification leading to a permanent alteration of protein function [52]. The server predicted amidation for its mutant residue cysteine, which can alter the localization and stability of the protein. It can also affect the sensitivity of the protein to surrounding pH, enhanced signaling, and binding to receptors [53]. R1158H had proteolytic cleavage prediction for both mutant and native residues, which does not vary, but cleavage in different positions in different cases may still change the type of alteration. Four SNPs (I109T, L411P, A401V, and R1158H) can lead to the altered metal-binding site according to MutPred2 server, which can be significant as this protein generally does not show the metal-binding property. A reason for the prediction can be their presence in N-terminal, C-terminal and catalytic domains of the protein. A gain of loop was seen for the substitution A816P which also has altered transmembrane function. A loop in structure can change the intrinsic functionality of protein along with their transmembrane property [54, 55]. A816P also gains relative solvent accessibility becoming exposed from buried, making it more available to have active site activity [56]. Altered transmembrane property was also predicted with substitutions G493D, P1152A and R1158H. G493D had a loss of strand and a gain of loop property, which can explain this [54, 55]. Y210C can lose its function with the loss of phosphorylation sites.

Conservation analysis further confirmed the pathogenicity of eight SNPs with high conservation score. Solvent accessibility analysis showed that L411P, G493D, A401V and L455F substitutions are both highly conserved and buried. Buried residues are generally located in the core protein, and substitution in them affects the protein function mostly [56].

Homology modeling was done with a template. Then the wild type structure was used to calculate RMSD value and TM align value to check the change in the 3D protein structures among the wild type and mutant residues. A higher RMSD value indicates more deviation in the structure between wild type and mutant protein structures [39]. TM align values of >0.5 and <1 show dissimilarity in the structures. Three substitutions G493D, L411P and L455F were finalized for further structural analysis by BIOVIA discovery studio visualizer according to these data. G493D and L411P showed a considerable number of changes in hydrogen bonds (Table 7). L411P and L455F were predicted to have a somatic mutation cluster according to Mutation 3D tool.

G493D is part of the PH2 domain, and glycine is changed to aspartic acid. Glycine is a strong hydrophilic amino acid, and analysis showed the change in hydrophobicity while it converts to aspartic acid (Table 8). Strong hydrophobicity can induce a change in binding capacity and interaction of the protein with other molecules [56, 57]. There is an increase in the number of hydrogen bonds, which can be the reason for a change in the free energy value, thus changing protein stability [51]. In the glycine residue, there are two hydrogen bonds with phenylalanine with a distance of 2.87Å and 2.94 Å (Fig 4a). These distances are 2.86Å and 3.01Å, respectively, with the mutant residue aspartic acid, which will indeed affect the Gibbs free energy [51]. Two extra hydrogen bonds are created with isoleucine and tryptophan (Fig 4b). The two new bonds formed have a distance of 2.72Å (isoleucine) and 2.9Å (tryptophan) from glycine. L411P mutation showed the amino acid change from leucine to proline, indicating a huge change in hydrophobicity (Table 8). Being changed from hydrophobic to hydrophilic can be a reason for significant structural change in the protein. A decrease of alkyl hydrophobic bond can be a reason behind this (Fig 5). Also, a leucine-leucine hydrogen bond is lost, decreasing the number of hydrogen bonds from two to one (Fig 5). The distances of other hydrogen bonds with lysine residue are 2.84Å (native) and 2.96Å (mutant). Finally, in the L455F substitution, one hydrogen bond increases with the mutation (Fig 6). Bond formation with lysine and serine remains the same with the distance of 3.24 Å and 3.26 Å in the wild type respectively and 3.34 Å and 3.17 Å in the mutant structure respectively. A new bond with phenylalanine is seen (3.17 Å). There is an apparent change in the angles of all the hydrogen bonds shown (Fig 7). As L411P and L455F are from the catalytic domain, these SNPs can be highly damaging to protein function as well.

**Fig 8:**
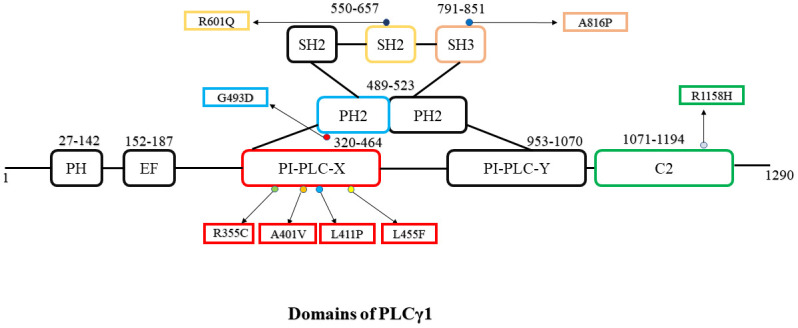
Domain organization with structural insights of PLCγ1 protein (protein ID ENSP00000362368). The final 8 nsSNPs shortlisted for structural analysis are marked in their domain.

GENEMANIA results showed *FYN* and *ITK* genes interact with *PLCG1*. These genes are involved in the T cell mediated pathways, the same as *PLCG1* and are related to diseases like Adult T cell leukemia [58, 59]. Involvement of these two genes with *PLCG1* can be a subject for further study. The results also showed that *PLCG1* shares domains with the following genes: *ITK*, *CBL*, *PLCG2*, *FYN* and *HCK*, which may allow them to offer similar functions. PH domain is common between (PLCG1 and ITK proteins which show interactions in the GENEMANIA analysis (S2 Fig). This conserved mammalian domain is responsible for the interaction between ITK and phosphoinositide 3-kinase (PI 3-kinase, PI3K) which in turn is the key player in lymphocyte differentiation and activation [60]. Computationally predicted functionally and structurally deleterious SNPs located in these regions could thus play an important role in this interaction (Fig 8). Protein-Protein interaction was seen between the SH2 domain of PLCG1 and ERBB2 which regulates protein tyrosine kinase. Catalytic domain PI-PLC-X box domain is also seen in PLCL1 protein which monitors GABA mediated synaptic inhibition [61]. FGFR1 which showed all possible interactions in GENEMANIA network with PLCG1, executes an engrossing complex activity with PLCG1. Through the binding of SH2, C2, and catalytic domain, they upregulate the status of these two proteins [62]. Our *in silico* analysis found that four SNPs located at the PI-PLC-X box domain (R355C, A401V, L411P and L455F, Fig 8) are functionally and structurally deleterious. Thus, these SNPs could potentially impact its functions. However, these findings should further be validated in laboratory experiments.

Among the non-coding SNPs, rs139043247 and rs543804707 showed the best result according to Regulome DB. They had a prediction of transcription binding sites, matched or unmatched motifs, and DNase footprint with DNase peak. rs139043247 also showed a significant result in the PolymiRTS database. Generally, the D and C classes with high conservation score and negative context score are the ones with the highest functional probable effect. Class D means the derived allele is disrupting a conserved site where class C means the creation of a new site [46]. This means there are high chances that the two SNPs rs139043247 and rs62621919 will affect the miRNA with probable mutations occurring in DNA.

## Conclusion

Out of all the missense SNPs, 16 SNPs were found to have deleterious effects by SIFT, PolyPhen-2, PROVEAN, and PANTHER tools. Further, 13 SNPs were predicted disease associated with disease predicting tools- PhD-SNP, Pmut and *SNPS&GO*. Ten SNPs were predicted to decrease the stability of the protein. Six SNPs (L411P, R355C, G493D, R1158H, A401V, L455F) were predicted highly conserved. Among them, L411P, G493D, A401V, L455F were predicted as the most significant ones with possible structural effect. Two mutations Y210C and R1158H had post-translational modification prediction in both wild type and mutant residues. Three SNPs L411P, G493D and L455F showed a promising structural change in the protein structure. R355C and R601Q mutations can also be important as they are part of domains that have shown previous relations with diseases. Among the non-coding region SNPs, rs139043247, rs543804707, and rs62621919 showed possible pathogenicity to interact with certain diseases and affect the functions of miRNAs. Further study of the gene *PLCG1* is highly necessary with the help of the data generated from the current study. The mentioned SNPs can be related to specific diseases mentioned earlier, especially with specific types which have been found related to the gene. Nevertheless, this is a computational study, and there will always be limitations regarding the analysis. So, there needs to be more *in vivo* researches with these data to prove their authenticity. Albeit, the study provided salient information by shedding light on the high-risk coding and non-coding SNPs of the target *PLCG1* gene to predict the possible diseases associated with the gene which will eventually help the researchers to find out a proper treatment plan to cure the disease-associated conditions.

## Supporting information

S1 Table Results of SIFT, PROVEAN, Ployphen-2 and PANTHER. (DOCX)

S2 Table Mutpred Results of the SNPs. (DOCX)

S3 Table Pmut, PhD-SNP, SNPS & GO Results. (DOCX)

S4 Table SNPs and INDELs in miRNA target sites from CLASH data (PolymiRTS). (DOCX)

S5 Table SNPs and INDELs in miRNA target sites (PolymiRTS). (DOCX)

S6 Table Target sites created by SNPs and INDELs in miRNA seeds (PolymiRTS). (DOCX)

S7 Table Regulome DB result. (DOCX)

S1 Fig Conservation scale data of Consurf. (TIF)

S2 Fig Gene-Gene interaction of PLCG1 Gene with different colors showing different types of interactions. (TIF)

S3 Fig Ramachandran Plot provided by Procheck for A401V mutation. (TIF)

S4 Fig Ramachandran Plot provided by Procheck for A816P mutation. (TIF)

S5 Fig Ramachandran Plot provided by Procheck for G493D mutation. (TIF)

S6 Fig Ramachandran Plot provided by Procheck for L411P mutation. (TIF)

S7 Fig Ramachandran Plot provided by Procheck for L455F mutation. (TIF)

S8 Fig Ramachandran Plot provided by Procheck for R355C mutation. (TIF)

S9 Fig Ramachandran Plot provided by Procheck for R601Q mutation. (TIF)

S10 Fig Ramachandran Plot provided by Procheck for R1158H mutation. (TIF)

S11 Fig Ramachandran Plot provided by Procheck for wild type protein structure. (TIF)
