# Supplementary material for: A computational in silico approach to predict high-risk coding and non-coding SNPs of human PLCG1 gene
Source: PLoS One. 2021 Nov 18;16(11):e0260054. doi: 10.1371/journal.pone.0260054 (PMC8601573; doi:10.1371/journal.pone.0260054)
Supplement: S1 Table — (DOCX) [file pone.0260054.s001.docx]

**Supporting Information**

**S1 Table 1. Results of SIFT, PROVEAN, Ployphen-2 and PANTHER**

| **SNP** | **Amino Acid Change** | **SIFT Prediction** | **SIFT Score** | **PROVEAN** | **PROVEAN Score** | **PolyPhen 2** | **Probability score** | **Panther** |
| --- | --- | --- | --- | --- | --- | --- | --- | --- |
| rs377574055 | R195Q | DELETERIOUS | 0.028 | N | -2.281 | possibly  damaging | 0.874 | probably damaging |
| rs373972267 | L411P | DELETERIOUS | 0.002 | D | -6.863 | probably damaging | 1 | probably damaging |
| rs370331909 | L37F | DELETERIOUS | 0.049 | N | -1.778 | possibly  damaging | 0.823 | probably damaging |
| rs369773324 | R974C | DELETERIOUS | 0.047 | N | -1.228 | probably damaging | 0.991 | possibly  damaging |
| rs367808225 | I109T | DELETERIOUS | 0.002 | D | -4.06 | probably damaging | 0.971 | probably damaging |
| rs202246756 | A816P | DELETERIOUS | 0.005 | D | -4.497 | probably damaging | 1 | probably damaging |
| rs201158224 | R355C | DELETERIOUS | 0.02 | D | -7.485 | probably damaging | 1 | probably damaging |
| rs200946488 | R601Q | DELETERIOUS | 0.032 | D | -3.413 | probably damaging | 1 | probably damaging |
| rs200475218 | R1083C | DELETERIOUS | 0.049 | D | -2.575 | benign | 0.038 | probably damaging |
| rs199826230 | Y210C | DELETERIOUS | 0.003 | D | -5.022 | probably damaging | 0.991 | possibly  damaging |
| rs199669312 | P244L | DELETERIOUS | 0.036 | D | -3.398 | possibly  damaging | 0.835 | possibly  damaging |
| rs191463364 | G493D | DELETERIOUS | 0.037 | D | -6.517 | probably damaging | 0.964 | probably damaging |
| rs186053167 | R1105L | DELETERIOUS | 0.004 | D | -6.414 | probably damaging | 0.995 | probably damaging |
| rs148020473 | P1152A | DELETERIOUS | 0.036 | D | -6.908 | probably damaging | 0.986 | probably damaging |
| rs147844565 | D1075V | DELETERIOUS | 0.031 | D | -7.077 | possibly  damaging | 0.919 | probably damaging |
| rs147137389 | S345C | DELETERIOUS | 0.007 | D | -3.82 | probably damaging | 1 | probably damaging |
| rs143441069 | I1114T | DELETERIOUS | 0.007 | D | -3.468 | benign | 0.26 | possibly  damaging |
| rs141684852 | R1158H | DELETERIOUS | 0 | D | -4.72 | probably damaging | 1 | probably damaging |
| rs7266677 | A401V | DELETERIOUS | 0.002 | D | -3.883 | probably damaging | 1 | probably damaging |
| rs6065316 | L455F | DELETERIOUS | 0.005 | D | -3.92 | probably damaging | 1 | probably damaging |
| rs2235361 | I949T | DELETERIOUS | 0.002 | D | -3.957 | probably damaging | 0.999 | probably damaging |
| rs375856103 | K536R | TOLERATED | 0.259 | N | -1.029 | benign | 0.002 | probably damaging |
| rs375791769 | R358Q | TOLERATED | 0.051 | D | -3.23 | probably damaging | 0.999 | probably damaging |
| rs375313716 | V317L | TOLERATED | 0.073 | N | -2.086 | benign | 0.172 | probably damaging |
| rs374561302 | T439I | TOLERATED | 0.116 | N | -2.44 | possibly  damaging | 0.877 | probably damaging |
| rs374424548 | A557V | TOLERATED | 0.057 | N | -1.787 | probably damaging | 0.969 | probably damaging |
| rs374417553 | Q1002R | TOLERATED | 0.571 | N | -0.056 | benign | 0.052 | probably damaging |
| rs372806807 | F254L | TOLERATED | 0.197 | D | -3.649 | possibly  damaging | 0.655 | probably damaging |
| rs372427036 | L673V | TOLERATED | 0.158 | N | -1.591 | possibly  damaging | 0.872 | probably damaging |
| rs372257814 | R1274Q | TOLERATED | 0.546 | N | -0.829 | benign | 0.052 | probably damaging |
| rs372179838 | N424D | TOLERATED | 0.495 | D | -2.62 | probably damaging | 0.971 | probably damaging |
| rs371242782 | V875F | TOLERATED | 0.392 | N | -0.831 | benign | 0.01 | probably damaging |
| rs371221687 | P852A | TOLERATED | 0.873 | N | -0.893 | benign | 0.001 | probably damaging |
| rs370818638 | R1083H | TOLERATED | 0.113 | N | -0.984 | benign | 0.038 | probably damaging |
| rs370780207 | A930T | TOLERATED | 0.833 | N | -0.718 | benign | 0.017 | probably damaging |
| rs370709447 | E758K | TOLERATED | 0.223 | N | -2.045 | possibly  damaging | 0.904 | probably damaging |
| rs370628843 | R197W | TOLERATED | 0.179 | D | -2.537 | probably damaging | 0.978 | possibly  damaging |
| rs369785655 | E537V | TOLERATED | 0.074 | D | -3.645 | benign | 0.149 | probably damaging |
| rs369565813 | V685L | TOLERATED | 0.171 | N | -2.108 | benign | 0.262 | probably damaging |
| rs367912334 | A671T | TOLERATED | 0.497 | N | -1.026 | possibly  damaging | 0.572 | probably damaging |
| rs367760831 | E864D | TOLERATED | 0.545 | N | 0.103 | benign | 0.019 | probably damaging |
| rs201939126 | R457K | TOLERATED | 0.587 | N | -1.664 | benign | 0.02 | probably damaging |
| rs201677341 | R1231Q | TOLERATED | 0.175 | N | -1.142 | probably damaging | 0.976 | probably damaging |
| rs201205160 | N728S | TOLERATED | 0.673 | N | 0.267 | benign | 0 | possibly  damaging |
| rs200825598 | F1260L | TOLERATED | 0.873 | N | 0.293 | benign | 0 | probably damaging |
| rs200775105 | I1209V | TOLERATED | 0.721 | N | -0.367 | benign | 0.017 | probably damaging |
| rs200504048 | S903L | TOLERATED | 0.318 | N | -2.305 | benign | 0.327 | possibly  damaging |
| rs200156118 | P284L | TOLERATED | 0.322 | D | -5.17 | benign | 0.082 | probably damaging |
| rs200112083 | G772R | TOLERATED | 0.156 | D | -3.391 | probably damaging | 0.974 | probably damaging |
| rs199859278 | R1243Q | TOLERATED | 0.578 | N | -0.038 | possibly  damaging | 0.929 | probably damaging |
| rs192373574 | R1252C | TOLERATED | 0.124 | N | -1.425 | probably damaging | 0.999 | probably damaging |
| rs192103227 | C881F | TOLERATED | 0.08 | D | -6.834 | probably damaging | 0.968 | probably damaging |
| rs181671926 | A671V | TOLERATED | 0.247 | N | -2.337 | possibly  damaging | 0.933 | probably damaging |
| rs180794782 | R248Q | TOLERATED | 0.579 | N | -0.1 | benign | 0.013 | possibly  damaging |
| rs151282703 | D914N | TOLERATED | 0.244 | N | -1.701 | benign | 0.035 | probably damaging |
| rs150665888 | R76H | TOLERATED | 0.185 | D | -3.554 | probably damaging | 0.999 | probably damaging |
| rs150381500 | P319L | TOLERATED | 0.653 | N | -2.435 | benign | 0.029 | probably damaging |
| rs146548575 | I574M | TOLERATED | 0.271 | N | -0.094 | benign | 0.002 | possibly  damaging |
| rs146160873 | L1220V | TOLERATED | 0.529 | N | 0.148 | benign | 0 | probably  benign |
| rs146143589 | V896I | TOLERATED | 0.26 | N | -0.503 | possibly damaging | 0.902 | probably damaging |
| rs145031121 | P252T | TOLERATED | 0.602 | N | 0.17 | benign | 0 | probably  benign |
| rs144676152 | A1244T | TOLERATED | 0.604 | N | 0.011 | benign | 0 | probably  benign |
| rs144195056 | R197Q | TOLERATED | 0.366 | N | -0.564 | possibly damaging | 0.458 | possibly  damaging |
| rs143541951 | P619L | TOLERATED | 0.623 | N | -1.923 | benign | 0.058 | probably damaging |
| rs142682393 | G721S | TOLERATED | 0.051 | D | -5.302 | probably damaging | 0.996 | probably damaging |
| rs142392437 | P476S | TOLERATED | 0.85 | N | 0.43 | benign | 0.001 | probably damaging |
| rs141297485 | T227M | TOLERATED | 0.228 | N | -0.46 | benign | 0.053 | possibly  damaging |
| rs140033921 | A902V | TOLERATED | 0.279 | N | -0.765 | benign | 0.003 | possibly  damaging |
| rs139300703 | Q916E | TOLERATED | 1 | N | -0.482 | benign | 0.001 | possibly  damaging |
| rs138431024 | T1185S | TOLERATED | 0.558 | N | -1.49 | benign | 0.376 | probably damaging |
| rs34203315 | S739T | TOLERATED | 0.212 | N | -1.853 | benign | 0.122 | probably damaging |
| rs2229348 | T209N | TOLERATED | 0.459 | N | 0.332 | benign | 0.001 | possibly  damaging |
| rs2228246 | S279G | TOLERATED | 0.412 | N | -1.263 | benign | 0 | probably  benign |
| rs753381 | I813T | TOLERATED | 1 | N | 1.037 | benign | 0 | probably  benign |
