# Supplementary material for: A computational in silico approach to predict high-risk coding and non-coding SNPs of human PLCG1 gene
Source: PLoS One. 2021 Nov 18;16(11):e0260054. doi: 10.1371/journal.pone.0260054 (PMC8601573; doi:10.1371/journal.pone.0260054)
Supplement: S2 Table — (DOCX) [file pone.0260054.s002.docx]

**S1 Table 2. Mutpred Results of the SNPs**

| **Amino Acid Change** | **Score** | **Modification** | **ELM** |
| --- | --- | --- | --- |
| L411P | 0.934 | Altered Metal binding (Pr = 0.24 \| P = 0.02); Altered Stability (Pr = 0.21 \| P = 0.01) | ELME000155\|ELME000333 |
| I109T | 0.815 | Altered Metal binding (Pr = 0.25 \| P = 0.04); Altered Stability (Pr = 0.19 \| P = 0.01) | ELME000337 |
| A816P | 0.907 | Altered Ordered interface (Pr = 0.31 \| P = 0.02); Gain of Loop (Pr = 0.27 \| P = 0.04); Altered Transmembrane protein (Pr = 0.25 \| P = 1.5e-03); Gain of Relative solvent accessibility (Pr = 0.25 \| P = 0.04) | ELME000085\|ELME000136\|ELME000159\|ELME000336 |
| R355C | 0.859 | Altered Ordered interface (Pr = 0.27 \| P = 8.3e-03) | ELME000106 |
| R601Q | 0.725 | Loss of Strand (Pr = 0.27 \| P = 0.02); Altered Ordered interface (Pr = 0.23 \| P = 0.05); Altered DNA binding (Pr = 0.23 \| P = 0.01) | None |
| Y210C | 0.577 | Loss of Phosphorylation at Y210 (Pr = 0.28 \| P = 0.02) | ELME000120\|PS00007 |
| G493D | 0.841 | Altered Transmembrane protein (Pr = 0.28 \| P = 4.8e-04); Gain of Helix (Pr = 0.27 \| P = 0.05); Loss of Strand (Pr = 0.26 \| P = 0.03) | ELME000335 |
| R1105L | 0.804 |  | ELME000316 |
| P1152A | 0.618 | Altered Transmembrane protein (Pr = 0.11 \| P = 0.03) | ELME000064\|ELME000122\|ELME000313\|ELME000336\|PS00006 |
| D1075V | 0.712 |  | ELME000107\|ELME000190 |
| R1158H | 0.796 | Altered Ordered interface (Pr = 0.28 \| P = 0.04); Loss of Strand (Pr = 0.26 \| P = 0.04); Altered Transmembrane protein (Pr = 0.25 \| P = 1.6e-03); Altered Metal binding (Pr = 0.23 \| P = 0.01); Gain of Sulfation at Y1162 (Pr = 0.14 \| P = 1.3e-03); Altered Stability (Pr = 0.10 \| P = 0.04) | ELME000300\|ELME000313 |
| A401V | 0.859 | Altered Metal binding (Pr = 0.29 \| P = 4.6e-03); Loss of Relative solvent accessibility (Pr = 0.28 \| P = 0.02) | ELME000193 |
| L455F | 0.799 | Gain of Strand (Pr = 0.27 \| P = 0.03); Gain of Acetylation at K456 (Pr = 0.19 \| P = 0.04) | None |
