# Supplementary material for: A computational in silico approach to predict high-risk coding and non-coding SNPs of human PLCG1 gene
Source: PLoS One. 2021 Nov 18;16(11):e0260054. doi: 10.1371/journal.pone.0260054 (PMC8601573; doi:10.1371/journal.pone.0260054)
Supplement: S3 Table — (DOCX) [file pone.0260054.s003.docx]

**S1 Table 3. Pmut, PhD-SNP, SNPS & GO Results**

| **SNP** | **Amino Acid Change** | **PMUT** | **Prediction Score** | **Percentage** | **PhD-SNP** | **RI** | **SNPS & Go** | **RI** | **PROBABILITY** |
| --- | --- | --- | --- | --- | --- | --- | --- | --- | --- |
| rs373972267 | L411P | Disease | 0.9279 | 94 | Disease | 9 | Disease | 6 | 0.816 |
| rs367808225 | I109T | Disease | 0.8768 | 92 | Disease | 8 | Disease | 2 | 0.622 |
| rs202246756 | A816P | Disease | 0.7257 | 87 | Disease | 4 | Disease | 2 | 0.584 |
| rs201158224 | R355C | Disease | 0.8656 | 91 | Disease | 7 | Disease | 6 | 0.8 |
| rs200946488 | R601Q | Disease | 0.6745 | 85 | Disease | 6 | Disease | 3 | 0.664 |
| rs199826230 | Y210C | Disease | 0.5806 | 82 | Disease | 7 | Disease | 6 | 0.797 |
| rs191463364 | G493D | Disease | 0.5223 | 79 | Disease | 6 | Disease | 0 | 0.502 |
| rs186053167 | R1105L | Disease | 0.6662 | 85 | Disease | 4 | Disease | 7 | 0.842 |
| rs148020473 | P1152A | Disease | 0.7901 | 89 | Disease | 6 | Disease | 2 | 0.615 |
| rs147844565 | D1075V | Disease | 0.7567 | 88 | Disease | 5 | Disease | 4 | 0.676 |
| rs141684852 | R1158H | Disease | 0.8341 | 90 | Disease | 9 | Disease | 5 | 0.745 |
| rs7266677 | A401V | Disease | 0.8669 | 91 | Disease | 8 | Disease | 6 | 0.817 |
| rs6065316 | L455F | Disease | 0.8526 | 91 | Disease | 8 | Disease | 2 | 0.583 |
| rs2235361 | I949T | Disease | 0.9122 | 93 | Neutral | 2 | Disease | 1 | 0.562 |
| rs199669312 | P244L | Disease | 0.5119 | 79 | Neutral | 4 | Neutral | 3 | 0.329 |
| rs147137389 | S345C | Neutral | 0.3785 | 87 | Neutral | 4 | Neutral | 2 | 0.413 |
