# Supplementary material for: A computational in silico approach to predict high-risk coding and non-coding SNPs of human PLCG1 gene
Source: PLoS One. 2021 Nov 18;16(11):e0260054. doi: 10.1371/journal.pone.0260054 (PMC8601573; doi:10.1371/journal.pone.0260054)
Supplement: S4 Table — (DOCX) [file pone.0260054.s004.docx]

**S1 Table 4*.* SNPs and INDELs in miRNA target sites from CLASH data (PolymiRTS)**

| **CLASH**  **SequenceID** | **miRNA** | **dbSNP ID** | **miRSite** | **Strand** |
| --- | --- | --- | --- | --- |
| L2HS-2765234_1 | [hsa-miR-1914*](http://www.mirbase.org/cgi-bin/query.pl?terms=hsa-miR-1914*&submit=Search) | [rs143541951](http://www.ncbi.nlm.nih.gov/SNP/snp_ref.cgi?rs=rs143541951) | ccgtatccaCTCCCGGcaagaTGCtGGGACCCCcaagttcttcttgacagacaacctcgtctt | + |
| L2HS-1363468_2 | [hsa-miR-744*](http://www.mirbase.org/cgi-bin/query.pl?terms=hsa-miR-744*&submit=Search) | [rs6072293](http://www.ncbi.nlm.nih.gov/SNP/snp_ref.cgi?rs=rs6072293) | gagcacAGGcTGAGcacatGCtaatGCGcgtccctcgtgatggggcc | + |
| L2HS-2765234_1 | [hsa-miR-1914*](http://www.mirbase.org/cgi-bin/query.pl?terms=hsa-miR-1914*&submit=Search) | [rs35256607](http://www.ncbi.nlm.nih.gov/SNP/snp_ref.cgi?rs=rs35256607) | ccgtatccaCTCCCGGcaagaTGCtGGGACCCCcaagttcttcttgacagacaacctcgtctt | + |
| L2-983871_1 | [hsa-miR-92a](http://www.mirbase.org/cgi-bin/query.pl?terms=hsa-miR-92a&submit=Search) | [rs142392437](http://www.ncbi.nlm.nih.gov/SNP/snp_ref.cgi?rs=rs142392437) | aagaaGCtGGCTGaGGGCAGTGCctacgaggaggtgcctacatccatgat | + |
| L2-983871_1 | [hsa-miR-92a](http://www.mirbase.org/cgi-bin/query.pl?terms=hsa-miR-92a&submit=Search) | [rs148776351](http://www.ncbi.nlm.nih.gov/SNP/snp_ref.cgi?rs=rs148776351) | aagaaGCtGGCTGaGGGCAGTGCctacgaggaggtgcctacatccatgat | + |
