# Supplementary material for: A computational in silico approach to predict high-risk coding and non-coding SNPs of human PLCG1 gene
Source: PLoS One. 2021 Nov 18;16(11):e0260054. doi: 10.1371/journal.pone.0260054 (PMC8601573; doi:10.1371/journal.pone.0260054)
Supplement: S5 Table — (DOCX) [file pone.0260054.s005.docx]

**S1 Table 5. SNPs and INDELs in miRNA target sites (PolymiRTS)**

| **Location** | **dbSNP ID** | **Variant**  **type** | **Wobble**  **base pair** | **Ancestral**  **Allele** | **Allele** | **miR ID** | **Conservation** | **miRSite** | **Function**  **Class** | **Exp**  **Support** | **context+**  **score change** |
| --- | --- | --- | --- | --- | --- | --- | --- | --- | --- | --- | --- |
| 39803182 | [rs62621919](http://www.ncbi.nlm.nih.gov/SNP/snp_ref.cgi?rs=rs62621919) | SNP | Y | G | G | [hsa-miR-3065-3p](http://www.mirbase.org/cgi-bin/mirna_entry.pl?acc=hsa-miR-3065-3p) | [8](http://compbio.uthsc.edu/miRSNP/miRSNP_detail_all.php) | gcaGGTGCTGtgc | D | N | -0.165 |
|  |  |  |  |  |  | [hsa-miR-6804-3p](http://www.mirbase.org/cgi-bin/mirna_entry.pl?acc=hsa-miR-6804-3p) | [6](http://compbio.uthsc.edu/miRSNP/miRSNP_detail_all.php) | gCAGGTGCtgtgc | D | N | -0.204 |
|  |  |  |  |  | A | [hsa-miR-101-3p](http://www.mirbase.org/cgi-bin/mirna_entry.pl?acc=hsa-miR-101-3p) | [4](http://compbio.uthsc.edu/miRSNP/miRSNP_detail_all.php) | gcagGTACTGTgc | C | N | -0.042 |
| 39803217 | [rs66814487](http://www.ncbi.nlm.nih.gov/SNP/snp_ref.cgi?rs=rs66814487) | INDEL | N | - | - | [hsa-miR-3133](http://www.mirbase.org/cgi-bin/mirna_entry.pl?acc=hsa-miR-3133) | [6](http://compbio.uthsc.edu/miRSNP/miRSNP_detail_all.php) | ggGTTCTTTggaa | O | N | 0.019 |
|  |  |  |  |  |  | [hsa-miR-548b-3p](http://www.mirbase.org/cgi-bin/mirna_entry.pl?acc=hsa-miR-548b-3p) | [11](http://compbio.uthsc.edu/miRSNP/miRSNP_detail_all.php) | gGGTTCTTtggaa | O | N | 0.022 |
|  |  |  |  |  | T |  |  |  |  |  |  |
| 39803222 | [rs150567235](http://www.ncbi.nlm.nih.gov/SNP/snp_ref.cgi?rs=rs150567235) | INDEL | N | - | - | [hsa-miR-2115-5p](http://www.mirbase.org/cgi-bin/mirna_entry.pl?acc=hsa-miR-2115-5p) | [2](http://compbio.uthsc.edu/miRSNP/miRSNP_detail_all.php) | tcttTGGAAGCAgcc | O | N | -0.321 |
|  |  |  |  |  |  | [hsa-miR-516a-3p](http://www.mirbase.org/cgi-bin/mirna_entry.pl?acc=hsa-miR-516a-3p) | [3](http://compbio.uthsc.edu/miRSNP/miRSNP_detail_all.php) | tctttGGAAGCAgcc | O | N | -0.107 |
|  |  |  |  |  |  | [hsa-miR-516b-3p](http://www.mirbase.org/cgi-bin/mirna_entry.pl?acc=hsa-miR-516b-3p) | [3](http://compbio.uthsc.edu/miRSNP/miRSNP_detail_all.php) | tctttGGAAGCAgcc | O | N | -0.107 |
|  |  |  |  |  |  | [hsa-miR-7162-5p](http://www.mirbase.org/cgi-bin/mirna_entry.pl?acc=hsa-miR-7162-5p) | [3](http://compbio.uthsc.edu/miRSNP/miRSNP_detail_all.php) | tctttGGAAGCAgcc | O | N | -0.107 |
|  |  |  |  |  | AG | [hsa-miR-1236-3p](http://www.mirbase.org/cgi-bin/mirna_entry.pl?acc=hsa-miR-1236-3p) | [3](http://compbio.uthsc.edu/miRSNP/miRSNP_detail_all.php) | tctttGGAAGAGcagcc | O | N | -0.132 |
| 39803224 | [rs35806184](http://www.ncbi.nlm.nih.gov/SNP/snp_ref.cgi?rs=rs35806184) | INDEL | N | - | - | [hsa-miR-2115-5p](http://www.mirbase.org/cgi-bin/mirna_entry.pl?acc=hsa-miR-2115-5p) | [2](http://compbio.uthsc.edu/miRSNP/miRSNP_detail_all.php) | ttTGGAAGCAgcccc | O | N | -0.321 |
|  |  |  |  |  |  | [hsa-miR-516a-3p](http://www.mirbase.org/cgi-bin/mirna_entry.pl?acc=hsa-miR-516a-3p) | [3](http://compbio.uthsc.edu/miRSNP/miRSNP_detail_all.php) | tttGGAAGCAgcccc | O | N | -0.107 |
|  |  |  |  |  |  | [hsa-miR-516b-3p](http://www.mirbase.org/cgi-bin/mirna_entry.pl?acc=hsa-miR-516b-3p) | [3](http://compbio.uthsc.edu/miRSNP/miRSNP_detail_all.php) | tttGGAAGCAgcccc | O | N | -0.107 |
|  |  |  |  |  |  | [hsa-miR-6762-3p](http://www.mirbase.org/cgi-bin/mirna_entry.pl?acc=hsa-miR-6762-3p) | [4](http://compbio.uthsc.edu/miRSNP/miRSNP_detail_all.php) | tttggaAGCAGCCcc | O | N | -0.026 |
|  |  |  |  |  |  | [hsa-miR-6829-5p](http://www.mirbase.org/cgi-bin/mirna_entry.pl?acc=hsa-miR-6829-5p) | [2](http://compbio.uthsc.edu/miRSNP/miRSNP_detail_all.php) | tttggaaGCAGCCCc | O | N | -0.017 |
|  |  |  |  |  |  | [hsa-miR-7162-5p](http://www.mirbase.org/cgi-bin/mirna_entry.pl?acc=hsa-miR-7162-5p) | [3](http://compbio.uthsc.edu/miRSNP/miRSNP_detail_all.php) | tttGGAAGCAgcccc | O | N | -0.107 |
|  |  |  |  |  | AG | [hsa-miR-1236-3p](http://www.mirbase.org/cgi-bin/mirna_entry.pl?acc=hsa-miR-1236-3p) | [3](http://compbio.uthsc.edu/miRSNP/miRSNP_detail_all.php) | tttGGAAGAGcagcccc | O | N | -0.132 |
| 39803328 | [rs35851917](http://www.ncbi.nlm.nih.gov/SNP/snp_ref.cgi?rs=rs35851917) | INDEL | N | - | - | [hsa-miR-3136-3p](http://www.mirbase.org/cgi-bin/mirna_entry.pl?acc=hsa-miR-3136-3p) | [10](http://compbio.uthsc.edu/miRSNP/miRSNP_detail_all.php) | ctgttTTGGGCCt | O | N | -0.258 |
|  |  |  |  |  |  | [hsa-miR-7155-3p](http://www.mirbase.org/cgi-bin/mirna_entry.pl?acc=hsa-miR-7155-3p) | [10](http://compbio.uthsc.edu/miRSNP/miRSNP_detail_all.php) | ctgttTTGGGCCt | O | N | -0.268 |
|  |  |  |  |  | G | [hsa-miR-5699-5p](http://www.mirbase.org/cgi-bin/mirna_entry.pl?acc=hsa-miR-5699-5p) | [10](http://compbio.uthsc.edu/miRSNP/miRSNP_detail_all.php) | ctgttTTGGGGCct | O | N | -0.244 |
|  |  |  |  |  |  | [hsa-miR-874-5p](http://www.mirbase.org/cgi-bin/mirna_entry.pl?acc=hsa-miR-874-5p) | [10](http://compbio.uthsc.edu/miRSNP/miRSNP_detail_all.php) | ctgtttTGGGGCCt | O | N | -0.322 |
| 39803329 | [rs66522560](http://www.ncbi.nlm.nih.gov/SNP/snp_ref.cgi?rs=rs66522560) | INDEL | N | - | - | [hsa-miR-3136-3p](http://www.mirbase.org/cgi-bin/mirna_entry.pl?acc=hsa-miR-3136-3p) | [10](http://compbio.uthsc.edu/miRSNP/miRSNP_detail_all.php) | tgttTTGGGCCtc | O | N | -0.258 |
|  |  |  |  |  |  | [hsa-miR-5008-5p](http://www.mirbase.org/cgi-bin/mirna_entry.pl?acc=hsa-miR-5008-5p) | [12](http://compbio.uthsc.edu/miRSNP/miRSNP_detail_all.php) | tgttttGGGCCTC | O | N | -0.041 |
|  |  |  |  |  |  | [hsa-miR-7155-3p](http://www.mirbase.org/cgi-bin/mirna_entry.pl?acc=hsa-miR-7155-3p) | [10](http://compbio.uthsc.edu/miRSNP/miRSNP_detail_all.php) | tgttTTGGGCCtc | O | N | -0.268 |
|  |  |  |  |  | G | [hsa-miR-5699-5p](http://www.mirbase.org/cgi-bin/mirna_entry.pl?acc=hsa-miR-5699-5p) | [10](http://compbio.uthsc.edu/miRSNP/miRSNP_detail_all.php) | tgttTTGGGGCctc | O | N | -0.244 |
|  |  |  |  |  |  | [hsa-miR-874-5p](http://www.mirbase.org/cgi-bin/mirna_entry.pl?acc=hsa-miR-874-5p) | [10](http://compbio.uthsc.edu/miRSNP/miRSNP_detail_all.php) | tgtttTGGGGCCtc | O | N | -0.322 |
| 39803334 | [rs66902410](http://www.ncbi.nlm.nih.gov/SNP/snp_ref.cgi?rs=rs66902410) | INDEL | N | - | - | [hsa-miR-3612](http://www.mirbase.org/cgi-bin/mirna_entry.pl?acc=hsa-miR-3612) | [8](http://compbio.uthsc.edu/miRSNP/miRSNP_detail_all.php) | tggGCCTCCAtgc | O | N | -0.17 |
|  |  |  |  |  |  | [hsa-miR-4443](http://www.mirbase.org/cgi-bin/mirna_entry.pl?acc=hsa-miR-4443) | [8](http://compbio.uthsc.edu/miRSNP/miRSNP_detail_all.php) | tggGCCTCCAtgc | O | N | -0.163 |
|  |  |  |  |  |  | [hsa-miR-4802-5p](http://www.mirbase.org/cgi-bin/mirna_entry.pl?acc=hsa-miR-4802-5p) | [5](http://compbio.uthsc.edu/miRSNP/miRSNP_detail_all.php) | tgggCCTCCATgc | O | N | -0.108 |
|  |  |  |  |  |  | [hsa-miR-5008-5p](http://www.mirbase.org/cgi-bin/mirna_entry.pl?acc=hsa-miR-5008-5p) | [12](http://compbio.uthsc.edu/miRSNP/miRSNP_detail_all.php) | tGGGCCTCcatgc | O | N | -0.304 |
|  |  |  |  |  |  | [hsa-miR-650](http://www.mirbase.org/cgi-bin/mirna_entry.pl?acc=hsa-miR-650) | [8](http://compbio.uthsc.edu/miRSNP/miRSNP_detail_all.php) | tggGCCTCCAtgc | O | N | -0.17 |
|  |  |  |  |  | T | [hsa-miR-3202](http://www.mirbase.org/cgi-bin/mirna_entry.pl?acc=hsa-miR-3202) | [5](http://compbio.uthsc.edu/miRSNP/miRSNP_detail_all.php) | tgggCCTTCCAtgc | O | N | -0.094 |
|  |  |  |  |  |  | [hsa-miR-4493](http://www.mirbase.org/cgi-bin/mirna_entry.pl?acc=hsa-miR-4493) | [12](http://compbio.uthsc.edu/miRSNP/miRSNP_detail_all.php) | tgGGCCTTCcatgc | O | N | -0.21 |
|  |  |  |  |  |  | [hsa-miR-4533](http://www.mirbase.org/cgi-bin/mirna_entry.pl?acc=hsa-miR-4533) | [5](http://compbio.uthsc.edu/miRSNP/miRSNP_detail_all.php) | tgggCCTTCCAtgc | O | N | -0.087 |
| 39803342 | [rs184484019](http://www.ncbi.nlm.nih.gov/SNP/snp_ref.cgi?rs=rs184484019) | SNP | N | C | C | [hsa-miR-4253](http://www.mirbase.org/cgi-bin/mirna_entry.pl?acc=hsa-miR-4253) | [3](http://compbio.uthsc.edu/miRSNP/miRSNP_detail_all.php) | CATGCCCcagctc | D | N | -0.181 |
|  |  |  |  |  |  | [hsa-miR-4487](http://www.mirbase.org/cgi-bin/mirna_entry.pl?acc=hsa-miR-4487) | [8](http://compbio.uthsc.edu/miRSNP/miRSNP_detail_all.php) | catgccCCAGCTC | D | N | -0.159 |
|  |  |  |  |  |  | [hsa-miR-4731-5p](http://www.mirbase.org/cgi-bin/mirna_entry.pl?acc=hsa-miR-4731-5p) | [4](http://compbio.uthsc.edu/miRSNP/miRSNP_detail_all.php) | catgCCCCAGCtc | D | N | -0.22 |
|  |  |  |  |  |  | [hsa-miR-486-3p](http://www.mirbase.org/cgi-bin/mirna_entry.pl?acc=hsa-miR-486-3p) | [4](http://compbio.uthsc.edu/miRSNP/miRSNP_detail_all.php) | caTGCCCCAgctc | D | N | -0.144 |
|  |  |  |  |  |  | [hsa-miR-6862-5p](http://www.mirbase.org/cgi-bin/mirna_entry.pl?acc=hsa-miR-6862-5p) | [3](http://compbio.uthsc.edu/miRSNP/miRSNP_detail_all.php) | CATGCCCcagctc | D | N | -0.171 |
|  |  |  |  |  | T | [hsa-miR-1827](http://www.mirbase.org/cgi-bin/mirna_entry.pl?acc=hsa-miR-1827) | [4](http://compbio.uthsc.edu/miRSNP/miRSNP_detail_all.php) | caTGCCTCAgctc | C | N | -0.086 |
|  |  |  |  |  |  | [hsa-miR-3160-3p](http://www.mirbase.org/cgi-bin/mirna_entry.pl?acc=hsa-miR-3160-3p) | [8](http://compbio.uthsc.edu/miRSNP/miRSNP_detail_all.php) | catgccTCAGCTC | C | N | -0.138 |
|  |  |  |  |  |  | [hsa-miR-4649-3p](http://www.mirbase.org/cgi-bin/mirna_entry.pl?acc=hsa-miR-4649-3p) | [3](http://compbio.uthsc.edu/miRSNP/miRSNP_detail_all.php) | catGCCTCAGctc | C | N | -0.198 |
|  |  |  |  |  |  | [hsa-miR-7160-5p](http://www.mirbase.org/cgi-bin/mirna_entry.pl?acc=hsa-miR-7160-5p) | [4](http://compbio.uthsc.edu/miRSNP/miRSNP_detail_all.php) | catgCCTCAGCtc | C | N | -0.186 |
| 39803452 | [rs188886607](http://www.ncbi.nlm.nih.gov/SNP/snp_ref.cgi?rs=rs188886607) | SNP | Y | G | G | [hsa-miR-4665-3p](http://www.mirbase.org/cgi-bin/mirna_entry.pl?acc=hsa-miR-4665-3p) | [4](http://compbio.uthsc.edu/miRSNP/miRSNP_detail_all.php) | atGGCCGAAgccc | D | N | -0.233 |
|  |  |  |  |  | A | [hsa-miR-3187-3p](http://www.mirbase.org/cgi-bin/mirna_entry.pl?acc=hsa-miR-3187-3p) | [3](http://compbio.uthsc.edu/miRSNP/miRSNP_detail_all.php) | ATGGCCAAagccc | C | N | -0.252 |
|  |  |  |  |  |  | [hsa-miR-4435](http://www.mirbase.org/cgi-bin/mirna_entry.pl?acc=hsa-miR-4435) | [3](http://compbio.uthsc.edu/miRSNP/miRSNP_detail_all.php) | aTGGCCAAagccc | C | N | -0.078 |
|  |  |  |  |  |  | [hsa-miR-4529-5p](http://www.mirbase.org/cgi-bin/mirna_entry.pl?acc=hsa-miR-4529-5p) | [3](http://compbio.uthsc.edu/miRSNP/miRSNP_detail_all.php) | ATGGCCAaagccc | C | N | -0.134 |
|  |  |  |  |  |  | [hsa-miR-4701-5p](http://www.mirbase.org/cgi-bin/mirna_entry.pl?acc=hsa-miR-4701-5p) | [3](http://compbio.uthsc.edu/miRSNP/miRSNP_detail_all.php) | aTGGCCAAagccc | C | N | -0.094 |
|  |  |  |  |  |  | [hsa-miR-548s](http://www.mirbase.org/cgi-bin/mirna_entry.pl?acc=hsa-miR-548s) | [3](http://compbio.uthsc.edu/miRSNP/miRSNP_detail_all.php) | aTGGCCAAagccc | C | N | -0.083 |
|  |  |  |  |  |  | [hsa-miR-588](http://www.mirbase.org/cgi-bin/mirna_entry.pl?acc=hsa-miR-588) | [3](http://compbio.uthsc.edu/miRSNP/miRSNP_detail_all.php) | aTGGCCAAagccc | C | N | -0.087 |
| 39803460 | [rs67893263](http://www.ncbi.nlm.nih.gov/SNP/snp_ref.cgi?rs=rs67893263) | INDEL | N | - | T | [hsa-miR-515-5p](http://www.mirbase.org/cgi-bin/mirna_entry.pl?acc=hsa-miR-515-5p) | [6](http://compbio.uthsc.edu/miRSNP/miRSNP_detail_all.php) | agccccTTGGAGA | O | N | 0.015 |
|  |  |  |  |  |  | [hsa-miR-519d-5p](http://www.mirbase.org/cgi-bin/mirna_entry.pl?acc=hsa-miR-519d-5p) | [6](http://compbio.uthsc.edu/miRSNP/miRSNP_detail_all.php) | agccccTTGGAGA | O | N | -0.012 |
|  |  |  |  |  |  | [hsa-miR-519e-5p](http://www.mirbase.org/cgi-bin/mirna_entry.pl?acc=hsa-miR-519e-5p) | [6](http://compbio.uthsc.edu/miRSNP/miRSNP_detail_all.php) | agccccTTGGAGA | O | N | 0.006 |
|  |  |  |  |  |  | [hsa-miR-5695](http://www.mirbase.org/cgi-bin/mirna_entry.pl?acc=hsa-miR-5695) | [6](http://compbio.uthsc.edu/miRSNP/miRSNP_detail_all.php) | agcccCTTGGAGA | O | N | -0.14 |
|  |  |  |  |  |  | [hsa-miR-6085](http://www.mirbase.org/cgi-bin/mirna_entry.pl?acc=hsa-miR-6085) | [11](http://compbio.uthsc.edu/miRSNP/miRSNP_detail_all.php) | AGCCCCTtggaga | O | N | -0.019 |
|  |  |  |  |  |  | [hsa-miR-6813-5p](http://www.mirbase.org/cgi-bin/mirna_entry.pl?acc=hsa-miR-6813-5p) | [11](http://compbio.uthsc.edu/miRSNP/miRSNP_detail_all.php) | AGCCCCTtggaga | O | N | -0.019 |
|  |  |  |  |  | - | [hsa-miR-3925-3p](http://www.mirbase.org/cgi-bin/mirna_entry.pl?acc=hsa-miR-3925-3p) | [6](http://compbio.uthsc.edu/miRSNP/miRSNP_detail_all.php) | agcccCTGGAGA | O | N | -0.062 |
|  |  |  |  |  |  | [hsa-miR-4685-5p](http://www.mirbase.org/cgi-bin/mirna_entry.pl?acc=hsa-miR-4685-5p) | [9](http://compbio.uthsc.edu/miRSNP/miRSNP_detail_all.php) | agcCCCTGGAga | O | N | -0.104 |
|  |  |  |  |  |  | [hsa-miR-6837-5p](http://www.mirbase.org/cgi-bin/mirna_entry.pl?acc=hsa-miR-6837-5p) | [9](http://compbio.uthsc.edu/miRSNP/miRSNP_detail_all.php) | agcCCCTGGAga | O | N | -0.104 |
|  |  |  |  |  |  | [hsa-miR-7113-5p](http://www.mirbase.org/cgi-bin/mirna_entry.pl?acc=hsa-miR-7113-5p) | [9](http://compbio.uthsc.edu/miRSNP/miRSNP_detail_all.php) | agcCCCTGGAga | O | N | -0.103 |
|  |  |  |  |  |  | [hsa-miR-766-3p](http://www.mirbase.org/cgi-bin/mirna_entry.pl?acc=hsa-miR-766-3p) | [6](http://compbio.uthsc.edu/miRSNP/miRSNP_detail_all.php) | agcccCTGGAGA | O | N | -0.043 |
| 39803483 | [rs141628590](http://www.ncbi.nlm.nih.gov/SNP/snp_ref.cgi?rs=rs141628590) | SNP | N | C | C | [hsa-miR-3064-5p](http://www.mirbase.org/cgi-bin/mirna_entry.pl?acc=hsa-miR-3064-5p) | [10](http://compbio.uthsc.edu/miRSNP/miRSNP_detail_all.php) | ctCAGCCAGtggc | D | N | -0.11 |
|  |  |  |  |  |  | [hsa-miR-4252](http://www.mirbase.org/cgi-bin/mirna_entry.pl?acc=hsa-miR-4252) | [10](http://compbio.uthsc.edu/miRSNP/miRSNP_detail_all.php) | ctcagcCAGTGGC | D | N | -0.184 |
|  |  |  |  |  |  | [hsa-miR-6504-5p](http://www.mirbase.org/cgi-bin/mirna_entry.pl?acc=hsa-miR-6504-5p) | [10](http://compbio.uthsc.edu/miRSNP/miRSNP_detail_all.php) | ctCAGCCAGtggc | D | N | -0.101 |
|  |  |  |  |  |  | [hsa-miR-892b](http://www.mirbase.org/cgi-bin/mirna_entry.pl?acc=hsa-miR-892b) | [10](http://compbio.uthsc.edu/miRSNP/miRSNP_detail_all.php) | ctcAGCCAGTggc | D | N | -0.075 |
|  |  |  |  |  | G | [hsa-miR-6825-3p](http://www.mirbase.org/cgi-bin/mirna_entry.pl?acc=hsa-miR-6825-3p) | [9](http://compbio.uthsc.edu/miRSNP/miRSNP_detail_all.php) | cTCAGCGAgtggc | C | N | -0.161 |
| 39803522 | [rs41283256](http://www.ncbi.nlm.nih.gov/SNP/snp_ref.cgi?rs=rs41283256) | SNP | Y | A |  |  |  |  |  |  |  |
|  |  |  |  |  | G | [hsa-miR-1266-5p](http://www.mirbase.org/cgi-bin/mirna_entry.pl?acc=hsa-miR-1266-5p) | [2](http://compbio.uthsc.edu/miRSNP/miRSNP_detail_all.php) | attCCTGAGAgtg | C | N | -0.079 |
|  |  |  |  |  |  | [hsa-miR-3664-3p](http://www.mirbase.org/cgi-bin/mirna_entry.pl?acc=hsa-miR-3664-3p) | [2](http://compbio.uthsc.edu/miRSNP/miRSNP_detail_all.php) | atTCCTGAGAgtg | C | N | -0.255 |
|  |  |  |  |  |  | [hsa-miR-4518](http://www.mirbase.org/cgi-bin/mirna_entry.pl?acc=hsa-miR-4518) | [2](http://compbio.uthsc.edu/miRSNP/miRSNP_detail_all.php) | attCCTGAGAgtg | C | N | -0.076 |
|  |  |  |  |  |  | [hsa-miR-4639-3p](http://www.mirbase.org/cgi-bin/mirna_entry.pl?acc=hsa-miR-4639-3p) | [3](http://compbio.uthsc.edu/miRSNP/miRSNP_detail_all.php) | attcctGAGAGTG | C | N | -0.11 |
|  |  |  |  |  |  | [hsa-miR-873-5p](http://www.mirbase.org/cgi-bin/mirna_entry.pl?acc=hsa-miR-873-5p) | [2](http://compbio.uthsc.edu/miRSNP/miRSNP_detail_all.php) | aTTCCTGAgagtg | C | N | -0.035 |
| 39803525 | [rs200348392](http://www.ncbi.nlm.nih.gov/SNP/snp_ref.cgi?rs=rs200348392) | INDEL | N | - | - | [hsa-miR-6777-3p](http://www.mirbase.org/cgi-bin/mirna_entry.pl?acc=hsa-miR-6777-3p) | [4](http://compbio.uthsc.edu/miRSNP/miRSNP_detail_all.php) | ttcctaAGAGTGGAgga | O | N | -0.131 |
|  |  |  |  |  | GTG |  |  |  |  |  |  |
| 39803528 | [rs35433692](http://www.ncbi.nlm.nih.gov/SNP/snp_ref.cgi?rs=rs35433692) | INDEL | N | - | - | [hsa-miR-4691-5p](http://www.mirbase.org/cgi-bin/mirna_entry.pl?acc=hsa-miR-4691-5p) | [4](http://compbio.uthsc.edu/miRSNP/miRSNP_detail_all.php) | ctaagagTGGAGGAgga | O | N | -0.022 |
|  |  |  |  |  |  | [hsa-miR-6777-3p](http://www.mirbase.org/cgi-bin/mirna_entry.pl?acc=hsa-miR-6777-3p) | [4](http://compbio.uthsc.edu/miRSNP/miRSNP_detail_all.php) | ctaAGAGTGGAggagga | O | N | -0.131 |
|  |  |  |  |  |  | [hsa-miR-6792-3p](http://www.mirbase.org/cgi-bin/mirna_entry.pl?acc=hsa-miR-6792-3p) | [4](http://compbio.uthsc.edu/miRSNP/miRSNP_detail_all.php) | ctaagagTGGAGGAgga | O | N | -0.013 |
|  |  |  |  |  | GTG |  |  |  |  |  |  |
| 39803530 | [rs114655995](http://www.ncbi.nlm.nih.gov/SNP/snp_ref.cgi?rs=rs114655995) | SNP | N | A | A | [hsa-miR-4691-5p](http://www.mirbase.org/cgi-bin/mirna_entry.pl?acc=hsa-miR-4691-5p) | [4](http://compbio.uthsc.edu/miRSNP/miRSNP_detail_all.php) | gagTGGAGGAgga | D | N | -0.019 |
|  |  |  |  |  |  | [hsa-miR-5193](http://www.mirbase.org/cgi-bin/mirna_entry.pl?acc=hsa-miR-5193) | [4](http://compbio.uthsc.edu/miRSNP/miRSNP_detail_all.php) | gagtgGAGGAGGA | D | N | -0.291 |
|  |  |  |  |  |  | [hsa-miR-660-3p](http://www.mirbase.org/cgi-bin/mirna_entry.pl?acc=hsa-miR-660-3p) | [4](http://compbio.uthsc.edu/miRSNP/miRSNP_detail_all.php) | gagtggAGGAGGA | D | N | -0.068 |
|  |  |  |  |  |  | [hsa-miR-6777-3p](http://www.mirbase.org/cgi-bin/mirna_entry.pl?acc=hsa-miR-6777-3p) | [4](http://compbio.uthsc.edu/miRSNP/miRSNP_detail_all.php) | GAGTGGAggagga | D | N | -0.133 |
|  |  |  |  |  |  | [hsa-miR-6792-3p](http://www.mirbase.org/cgi-bin/mirna_entry.pl?acc=hsa-miR-6792-3p) | [4](http://compbio.uthsc.edu/miRSNP/miRSNP_detail_all.php) | gagTGGAGGAgga | D | N | -0.01 |
|  |  |  |  |  |  |  |  |  |  |  |  |
| 39803604 | [rs146184925](http://www.ncbi.nlm.nih.gov/SNP/snp_ref.cgi?rs=rs146184925) | SNP | N | C | C | [hsa-miR-3620-3p](http://www.mirbase.org/cgi-bin/mirna_entry.pl?acc=hsa-miR-3620-3p) | [10](http://compbio.uthsc.edu/miRSNP/miRSNP_detail_all.php) | gctgggCAGGGTG | D | N | -0.162 |
|  |  |  |  |  |  | [hsa-miR-6752-3p](http://www.mirbase.org/cgi-bin/mirna_entry.pl?acc=hsa-miR-6752-3p) | [9](http://compbio.uthsc.edu/miRSNP/miRSNP_detail_all.php) | gctgGGCAGGGtg | D | N | -0.161 |
|  |  |  |  |  | T | [hsa-miR-1295b-5p](http://www.mirbase.org/cgi-bin/mirna_entry.pl?acc=hsa-miR-1295b-5p) | [5](http://compbio.uthsc.edu/miRSNP/miRSNP_detail_all.php) | gCTGGGTAgggtg | C | N | -0.111 |
|  |  |  |  |  |  | [hsa-miR-1912](http://www.mirbase.org/cgi-bin/mirna_entry.pl?acc=hsa-miR-1912) | [5](http://compbio.uthsc.edu/miRSNP/miRSNP_detail_all.php) | gCTGGGTAgggtg | C | N | -0.108 |
|  |  |  |  |  |  | [hsa-miR-3130-5p](http://www.mirbase.org/cgi-bin/mirna_entry.pl?acc=hsa-miR-3130-5p) | [5](http://compbio.uthsc.edu/miRSNP/miRSNP_detail_all.php) | gCTGGGTAgggtg | C | N | -0.128 |
|  |  |  |  |  |  | [hsa-miR-3162-3p](http://www.mirbase.org/cgi-bin/mirna_entry.pl?acc=hsa-miR-3162-3p) | [9](http://compbio.uthsc.edu/miRSNP/miRSNP_detail_all.php) | gctgGGTAGGGtg | C | N | -0.166 |
|  |  |  |  |  |  | [hsa-miR-4482-5p](http://www.mirbase.org/cgi-bin/mirna_entry.pl?acc=hsa-miR-4482-5p) | [5](http://compbio.uthsc.edu/miRSNP/miRSNP_detail_all.php) | gCTGGGTAgggtg | C | N | -0.118 |
| 39803716 | [rs139043247](http://www.ncbi.nlm.nih.gov/SNP/snp_ref.cgi?rs=rs139043247) | SNP | Y | G | G | [hsa-miR-6780b-3p](http://www.mirbase.org/cgi-bin/mirna_entry.pl?acc=hsa-miR-6780b-3p) | [14](http://compbio.uthsc.edu/miRSNP/miRSNP_detail_all.php) | gcACAAGGGcaca | D | N | -0.072 |
|  |  |  |  |  |  | [hsa-miR-6886-3p](http://www.mirbase.org/cgi-bin/mirna_entry.pl?acc=hsa-miR-6886-3p) | [13](http://compbio.uthsc.edu/miRSNP/miRSNP_detail_all.php) | gcacAAGGGCAca | D | N | -0.105 |
|  |  |  |  |  |  | [hsa-miR-938](http://www.mirbase.org/cgi-bin/mirna_entry.pl?acc=hsa-miR-938) | [13](http://compbio.uthsc.edu/miRSNP/miRSNP_detail_all.php) | gcacAAGGGCAca | D | N | -0.103 |
|  |  |  |  |  | A | [hsa-miR-1271-3p](http://www.mirbase.org/cgi-bin/mirna_entry.pl?acc=hsa-miR-1271-3p) | [10](http://compbio.uthsc.edu/miRSNP/miRSNP_detail_all.php) | gcacaaAGGCACA | C | N | -0.107 |
|  |  |  |  |  |  | [hsa-miR-2113](http://www.mirbase.org/cgi-bin/mirna_entry.pl?acc=hsa-miR-2113) | [14](http://compbio.uthsc.edu/miRSNP/miRSNP_detail_all.php) | GCACAAAggcaca | C | N | -0.034 |
|  |  |  |  |  |  | [hsa-miR-550a-3-5p](http://www.mirbase.org/cgi-bin/mirna_entry.pl?acc=hsa-miR-550a-3-5p) | [10](http://compbio.uthsc.edu/miRSNP/miRSNP_detail_all.php) | gcacaaAGGCACA | C | N | -0.093 |
|  |  |  |  |  |  | [hsa-miR-550a-5p](http://www.mirbase.org/cgi-bin/mirna_entry.pl?acc=hsa-miR-550a-5p) | [10](http://compbio.uthsc.edu/miRSNP/miRSNP_detail_all.php) | gcacaaAGGCACA | C | N | -0.093 |
|  |  |  |  |  |  | [hsa-miR-550b-2-5p](http://www.mirbase.org/cgi-bin/mirna_entry.pl?acc=hsa-miR-550b-2-5p) | [10](http://compbio.uthsc.edu/miRSNP/miRSNP_detail_all.php) | gcacaaAGGCACA | C | N | -0.15 |
| 39803791 | [rs184072600](http://www.ncbi.nlm.nih.gov/SNP/snp_ref.cgi?rs=rs184072600) | SNP | N | T | T | [hsa-miR-369-3p](http://www.mirbase.org/cgi-bin/mirna_entry.pl?acc=hsa-miR-369-3p) | [10](http://compbio.uthsc.edu/miRSNP/miRSNP_detail_all.php) | GTATTATttttcg | D | N | -0.051 |
|  |  |  |  |  |  | [hsa-miR-5692a](http://www.mirbase.org/cgi-bin/mirna_entry.pl?acc=hsa-miR-5692a) | [10](http://compbio.uthsc.edu/miRSNP/miRSNP_detail_all.php) | gtATTATTTttcg | D | N | 0.079 |
|  |  |  |  |  | C | [hsa-miR-548a-5p](http://www.mirbase.org/cgi-bin/mirna_entry.pl?acc=hsa-miR-548a-5p) | [10](http://compbio.uthsc.edu/miRSNP/miRSNP_detail_all.php) | gtaTTACTTTtcg | C | N | -0.036 |
|  |  |  |  |  |  | [hsa-miR-548ab](http://www.mirbase.org/cgi-bin/mirna_entry.pl?acc=hsa-miR-548ab) | [10](http://compbio.uthsc.edu/miRSNP/miRSNP_detail_all.php) | gtaTTACTTTtcg | C | N | -0.045 |
|  |  |  |  |  |  | [hsa-miR-548ak](http://www.mirbase.org/cgi-bin/mirna_entry.pl?acc=hsa-miR-548ak) | [10](http://compbio.uthsc.edu/miRSNP/miRSNP_detail_all.php) | gtaTTACTTTtcg | C | N | -0.045 |
|  |  |  |  |  |  | [hsa-miR-548am-5p](http://www.mirbase.org/cgi-bin/mirna_entry.pl?acc=hsa-miR-548am-5p) | [10](http://compbio.uthsc.edu/miRSNP/miRSNP_detail_all.php) | gtaTTACTTTtcg | C | N | -0.045 |
|  |  |  |  |  |  | [hsa-miR-548ap-5p](http://www.mirbase.org/cgi-bin/mirna_entry.pl?acc=hsa-miR-548ap-5p) | [10](http://compbio.uthsc.edu/miRSNP/miRSNP_detail_all.php) | gtaTTACTTTtcg | C | N | -0.008 |
|  |  |  |  |  |  | [hsa-miR-548aq-5p](http://www.mirbase.org/cgi-bin/mirna_entry.pl?acc=hsa-miR-548aq-5p) | [10](http://compbio.uthsc.edu/miRSNP/miRSNP_detail_all.php) | gtaTTACTTTtcg | C | N | -0.045 |
|  |  |  |  |  |  | [hsa-miR-548ar-5p](http://www.mirbase.org/cgi-bin/mirna_entry.pl?acc=hsa-miR-548ar-5p) | [10](http://compbio.uthsc.edu/miRSNP/miRSNP_detail_all.php) | gtaTTACTTTtcg | C | N | -0.045 |
|  |  |  |  |  |  | [hsa-miR-548as-5p](http://www.mirbase.org/cgi-bin/mirna_entry.pl?acc=hsa-miR-548as-5p) | [10](http://compbio.uthsc.edu/miRSNP/miRSNP_detail_all.php) | gtaTTACTTTtcg | C | N | -0.027 |
|  |  |  |  |  |  | [hsa-miR-548au-5p](http://www.mirbase.org/cgi-bin/mirna_entry.pl?acc=hsa-miR-548au-5p) | [10](http://compbio.uthsc.edu/miRSNP/miRSNP_detail_all.php) | gtaTTACTTTtcg | C | N | -0.045 |
|  |  |  |  |  |  | [hsa-miR-548ay-5p](http://www.mirbase.org/cgi-bin/mirna_entry.pl?acc=hsa-miR-548ay-5p) | [10](http://compbio.uthsc.edu/miRSNP/miRSNP_detail_all.php) | gtaTTACTTTtcg | C | N | -0.045 |
|  |  |  |  |  |  | [hsa-miR-548b-5p](http://www.mirbase.org/cgi-bin/mirna_entry.pl?acc=hsa-miR-548b-5p) | [10](http://compbio.uthsc.edu/miRSNP/miRSNP_detail_all.php) | gtaTTACTTTtcg | C | N | -0.036 |
|  |  |  |  |  |  | [hsa-miR-548c-5p](http://www.mirbase.org/cgi-bin/mirna_entry.pl?acc=hsa-miR-548c-5p) | [10](http://compbio.uthsc.edu/miRSNP/miRSNP_detail_all.php) | gtaTTACTTTtcg | C | N | -0.045 |
|  |  |  |  |  |  | [hsa-miR-548d-5p](http://www.mirbase.org/cgi-bin/mirna_entry.pl?acc=hsa-miR-548d-5p) | [10](http://compbio.uthsc.edu/miRSNP/miRSNP_detail_all.php) | gtaTTACTTTtcg | C | N | -0.045 |
|  |  |  |  |  |  | [hsa-miR-548h-5p](http://www.mirbase.org/cgi-bin/mirna_entry.pl?acc=hsa-miR-548h-5p) | [10](http://compbio.uthsc.edu/miRSNP/miRSNP_detail_all.php) | gtaTTACTTTtcg | C | N | -0.045 |
|  |  |  |  |  |  | [hsa-miR-548i](http://www.mirbase.org/cgi-bin/mirna_entry.pl?acc=hsa-miR-548i) | [10](http://compbio.uthsc.edu/miRSNP/miRSNP_detail_all.php) | gtaTTACTTTtcg | C | N | -0.045 |
|  |  |  |  |  |  | [hsa-miR-548j-5p](http://www.mirbase.org/cgi-bin/mirna_entry.pl?acc=hsa-miR-548j-5p) | [10](http://compbio.uthsc.edu/miRSNP/miRSNP_detail_all.php) | gtaTTACTTTtcg | C | N | -0.008 |
|  |  |  |  |  |  | [hsa-miR-548n](http://www.mirbase.org/cgi-bin/mirna_entry.pl?acc=hsa-miR-548n) | [11](http://compbio.uthsc.edu/miRSNP/miRSNP_detail_all.php) | gtatTACTTTTcg | C | N | -0.018 |
|  |  |  |  |  |  | [hsa-miR-548o-5p](http://www.mirbase.org/cgi-bin/mirna_entry.pl?acc=hsa-miR-548o-5p) | [10](http://compbio.uthsc.edu/miRSNP/miRSNP_detail_all.php) | gtaTTACTTTtcg | C | N | -0.045 |
|  |  |  |  |  |  | [hsa-miR-548w](http://www.mirbase.org/cgi-bin/mirna_entry.pl?acc=hsa-miR-548w) | [10](http://compbio.uthsc.edu/miRSNP/miRSNP_detail_all.php) | gtaTTACTTTtcg | C | N | -0.045 |
|  |  |  |  |  |  | [hsa-miR-548y](http://www.mirbase.org/cgi-bin/mirna_entry.pl?acc=hsa-miR-548y) | [10](http://compbio.uthsc.edu/miRSNP/miRSNP_detail_all.php) | gtaTTACTTTtcg | C | N | -0.045 |
|  |  |  |  |  |  | [hsa-miR-559](http://www.mirbase.org/cgi-bin/mirna_entry.pl?acc=hsa-miR-559) | [10](http://compbio.uthsc.edu/miRSNP/miRSNP_detail_all.php) | gtaTTACTTTtcg | C | N | -0.027 |
| 39803803 | [rs77819029](http://www.ncbi.nlm.nih.gov/SNP/snp_ref.cgi?rs=rs77819029) | SNP | Y | A | A | [hsa-miR-3672](http://www.mirbase.org/cgi-bin/mirna_entry.pl?acc=hsa-miR-3672) | [1](http://compbio.uthsc.edu/miRSNP/miRSNP_detail_all.php) | GTCTCAAtggtat | N | N | -0.121 |
|  |  |  |  |  |  | [hsa-miR-4461](http://www.mirbase.org/cgi-bin/mirna_entry.pl?acc=hsa-miR-4461) | [5](http://compbio.uthsc.edu/miRSNP/miRSNP_detail_all.php) | gTCTCAATggtat | D | N | -0.06 |
|  |  |  |  |  |  | [hsa-miR-6512-5p](http://www.mirbase.org/cgi-bin/mirna_entry.pl?acc=hsa-miR-6512-5p) | [5](http://compbio.uthsc.edu/miRSNP/miRSNP_detail_all.php) | gtctcAATGGTAt | D | N | -0.072 |
|  |  |  |  |  |  | [hsa-miR-6864-3p](http://www.mirbase.org/cgi-bin/mirna_entry.pl?acc=hsa-miR-6864-3p) | [1](http://compbio.uthsc.edu/miRSNP/miRSNP_detail_all.php) | GTCTCAAtggtat | N | N | -0.107 |
|  |  |  |  |  | G | [hsa-miR-1255b-2-3p](http://www.mirbase.org/cgi-bin/mirna_entry.pl?acc=hsa-miR-1255b-2-3p) | [5](http://compbio.uthsc.edu/miRSNP/miRSNP_detail_all.php) | gtctcAGTGGTAt | C | N | -0.122 |
|  |  |  |  |  |  | [hsa-miR-181a-2-3p](http://www.mirbase.org/cgi-bin/mirna_entry.pl?acc=hsa-miR-181a-2-3p) | [5](http://compbio.uthsc.edu/miRSNP/miRSNP_detail_all.php) | gtcTCAGTGGtat | C | N | -0.177 |
|  |  |  |  |  |  | [hsa-miR-4329](http://www.mirbase.org/cgi-bin/mirna_entry.pl?acc=hsa-miR-4329) | [1](http://compbio.uthsc.edu/miRSNP/miRSNP_detail_all.php) | GTCTCAGtggtat | C | N | -0.135 |
| 39803829 | [rs34400187](http://www.ncbi.nlm.nih.gov/SNP/snp_ref.cgi?rs=rs34400187) | INDEL | N | - | CCT | [hsa-miR-4459](http://www.mirbase.org/cgi-bin/mirna_entry.pl?acc=hsa-miR-4459) | [6](http://compbio.uthsc.edu/miRSNP/miRSNP_detail_all.php) | tatttactCCTCCTGct | O | N | -0.133 |
|  |  |  |  |  |  | [hsa-miR-765](http://www.mirbase.org/cgi-bin/mirna_entry.pl?acc=hsa-miR-765) | [5](http://compbio.uthsc.edu/miRSNP/miRSNP_detail_all.php) | tatttaCTCCTCCtgct | O | N | -0.193 |
|  |  |  |  |  | - | [hsa-miR-4433-3p](http://www.mirbase.org/cgi-bin/mirna_entry.pl?acc=hsa-miR-4433-3p) | [5](http://compbio.uthsc.edu/miRSNP/miRSNP_detail_all.php) | tatttACTCCTGct | O | N | -0.112 |
|  |  |  |  |  |  | [hsa-miR-4722-5p](http://www.mirbase.org/cgi-bin/mirna_entry.pl?acc=hsa-miR-4722-5p) | [5](http://compbio.uthsc.edu/miRSNP/miRSNP_detail_all.php) | tatttaCTCCTGCt | O | N | -0.056 |
|  |  |  |  |  |  | [hsa-miR-6165](http://www.mirbase.org/cgi-bin/mirna_entry.pl?acc=hsa-miR-6165) | [5](http://compbio.uthsc.edu/miRSNP/miRSNP_detail_all.php) | tatttacTCCTGCT | O | N | -0.007 |
| 39803976 | [rs6072297](http://www.ncbi.nlm.nih.gov/SNP/snp_ref.cgi?rs=rs6072297) | SNP | N | C | C | [hsa-miR-760](http://www.mirbase.org/cgi-bin/mirna_entry.pl?acc=hsa-miR-760) | [3](http://compbio.uthsc.edu/miRSNP/miRSNP_detail_all.php) | CAGAGCCtatttt | D | N | -0.209 |
|  |  |  |  |  | T | [hsa-miR-6796-3p](http://www.mirbase.org/cgi-bin/mirna_entry.pl?acc=hsa-miR-6796-3p) | [1](http://compbio.uthsc.edu/miRSNP/miRSNP_detail_all.php) | cAGAGCTTAtttt | C | N | -0.261 |
|  |  |  |  |  |  | [hsa-miR-99a-3p](http://www.mirbase.org/cgi-bin/mirna_entry.pl?acc=hsa-miR-99a-3p) | [1](http://compbio.uthsc.edu/miRSNP/miRSNP_detail_all.php) | caGAGCTTAtttt | C | N | -0.156 |
|  |  |  |  |  |  | [hsa-miR-99b-3p](http://www.mirbase.org/cgi-bin/mirna_entry.pl?acc=hsa-miR-99b-3p) | [1](http://compbio.uthsc.edu/miRSNP/miRSNP_detail_all.php) | caGAGCTTAtttt | C | N | -0.143 |
| 39804061 | [rs6065319](http://www.ncbi.nlm.nih.gov/SNP/snp_ref.cgi?rs=rs6065319) | SNP | N | C | C | [hsa-miR-4269](http://www.mirbase.org/cgi-bin/mirna_entry.pl?acc=hsa-miR-4269) | [1](http://compbio.uthsc.edu/miRSNP/miRSNP_detail_all.php) | agtTGCCTGActc | N | N | -0.094 |
|  |  |  |  |  |  | [hsa-miR-4514](http://www.mirbase.org/cgi-bin/mirna_entry.pl?acc=hsa-miR-4514) | [1](http://compbio.uthsc.edu/miRSNP/miRSNP_detail_all.php) | agtTGCCTGActc | N | N | -0.072 |
|  |  |  |  |  |  | [hsa-miR-4692](http://www.mirbase.org/cgi-bin/mirna_entry.pl?acc=hsa-miR-4692) | [1](http://compbio.uthsc.edu/miRSNP/miRSNP_detail_all.php) | agtTGCCTGActc | N | N | -0.079 |
|  |  |  |  |  |  | [hsa-miR-4742-5p](http://www.mirbase.org/cgi-bin/mirna_entry.pl?acc=hsa-miR-4742-5p) | [1](http://compbio.uthsc.edu/miRSNP/miRSNP_detail_all.php) | agTTGCCTGActc | N | N | -0.228 |
|  |  |  |  |  |  | [hsa-miR-5702](http://www.mirbase.org/cgi-bin/mirna_entry.pl?acc=hsa-miR-5702) | [1](http://compbio.uthsc.edu/miRSNP/miRSNP_detail_all.php) | agttgcCTGACTC | N | N | -0.089 |
|  |  |  |  |  |  | [hsa-miR-6715b-5p](http://www.mirbase.org/cgi-bin/mirna_entry.pl?acc=hsa-miR-6715b-5p) | [1](http://compbio.uthsc.edu/miRSNP/miRSNP_detail_all.php) | agtTGCCTGActc | N | N | -0.088 |
|  |  |  |  |  |  |  |  |  |  |  |  |
| 39804094 | [rs6102297](http://www.ncbi.nlm.nih.gov/SNP/snp_ref.cgi?rs=rs6102297) | SNP | Y | G | G | [hsa-miR-3065-3p](http://www.mirbase.org/cgi-bin/mirna_entry.pl?acc=hsa-miR-3065-3p) | [21](http://compbio.uthsc.edu/miRSNP/miRSNP_detail_all.php) | cagttaGGTGCTG | D | N | -0.274 |
|  |  |  |  |  | A | [hsa-miR-383-3p](http://www.mirbase.org/cgi-bin/mirna_entry.pl?acc=hsa-miR-383-3p) | [21](http://compbio.uthsc.edu/miRSNP/miRSNP_detail_all.php) | cagttaAGTGCTG | C | N | -0.209 |
| 39804238 | [rs150066829](http://www.ncbi.nlm.nih.gov/SNP/snp_ref.cgi?rs=rs150066829) | SNP | N | G | G | [hsa-miR-31-3p](http://www.mirbase.org/cgi-bin/mirna_entry.pl?acc=hsa-miR-31-3p) | [8](http://compbio.uthsc.edu/miRSNP/miRSNP_detail_all.php) | ctcataGCATAGC | D | N | -0.31 |
|  |  |  |  |  |  | [hsa-miR-3146](http://www.mirbase.org/cgi-bin/mirna_entry.pl?acc=hsa-miR-3146) | [4](http://compbio.uthsc.edu/miRSNP/miRSNP_detail_all.php) | ctcaTAGCATAgc | D | N | -0.057 |
|  |  |  |  |  | C | [hsa-miR-5579-5p](http://www.mirbase.org/cgi-bin/mirna_entry.pl?acc=hsa-miR-5579-5p) | [4](http://compbio.uthsc.edu/miRSNP/miRSNP_detail_all.php) | ctcaTACCATAgc | C | N | -0.052 |
| 39804288 | [rs6072298](http://www.ncbi.nlm.nih.gov/SNP/snp_ref.cgi?rs=rs6072298) | SNP | Y | G | G | [hsa-miR-1199-5p](http://www.mirbase.org/cgi-bin/mirna_entry.pl?acc=hsa-miR-1199-5p) | [8](http://compbio.uthsc.edu/miRSNP/miRSNP_detail_all.php) | GGCTCAGggttgg | D | N | -0.186 |
|  |  |  |  |  |  | [hsa-miR-125a-5p](http://www.mirbase.org/cgi-bin/mirna_entry.pl?acc=hsa-miR-125a-5p) | [8](http://compbio.uthsc.edu/miRSNP/miRSNP_detail_all.php) | ggCTCAGGGttgg | D | N | -0.167 |
|  |  |  |  |  |  | [hsa-miR-125b-5p](http://www.mirbase.org/cgi-bin/mirna_entry.pl?acc=hsa-miR-125b-5p) | [8](http://compbio.uthsc.edu/miRSNP/miRSNP_detail_all.php) | ggCTCAGGGttgg | D | N | -0.176 |
|  |  |  |  |  |  | [hsa-miR-4319](http://www.mirbase.org/cgi-bin/mirna_entry.pl?acc=hsa-miR-4319) | [8](http://compbio.uthsc.edu/miRSNP/miRSNP_detail_all.php) | ggCTCAGGGttgg | D | N | -0.176 |
|  |  |  |  |  |  | [hsa-miR-6732-3p](http://www.mirbase.org/cgi-bin/mirna_entry.pl?acc=hsa-miR-6732-3p) | [8](http://compbio.uthsc.edu/miRSNP/miRSNP_detail_all.php) | ggctCAGGGTTgg | D | N | -0.148 |
|  |  |  |  |  |  | [hsa-miR-6751-3p](http://www.mirbase.org/cgi-bin/mirna_entry.pl?acc=hsa-miR-6751-3p) | [8](http://compbio.uthsc.edu/miRSNP/miRSNP_detail_all.php) | GGCTCAGggttgg | D | N | -0.177 |
|  |  |  |  |  | A | [hsa-miR-6807-5p](http://www.mirbase.org/cgi-bin/mirna_entry.pl?acc=hsa-miR-6807-5p) | [8](http://compbio.uthsc.edu/miRSNP/miRSNP_detail_all.php) | GGCTCAAggttgg | C | N | -0.159 |
