# Supplementary material for: A computational in silico approach to predict high-risk coding and non-coding SNPs of human PLCG1 gene
Source: PLoS One. 2021 Nov 18;16(11):e0260054. doi: 10.1371/journal.pone.0260054 (PMC8601573; doi:10.1371/journal.pone.0260054)
Supplement: S6 Table — (DOCX) [file pone.0260054.s006.docx]

| **S1 Table 6. Target sites created by SNPs and INDELs in miRNA seeds (PolymiRTS)** | | | | | | | | |
| --- | --- | --- | --- | --- | --- | --- | --- | --- |
| **Location** | **miR ID** | **dbSNP ID** | **miR Seed** | **Allele** | **Wobble base pair** | **miRSite** | **Conservation** | **context+ score change** |
| 39803206 | [hsa-miR-671-3p](http://microrna.sanger.ac.uk/cgi-bin/sequences/mirna_entry.pl?acc=) | [rs200733440](http://www.ncbi.nlm.nih.gov/SNP/snp_ref.cgi?rs=rs200733440) | CC[G/A]GUUC | G/A | 1 | GAACUGG | [9](http://compbio.uthsc.edu/miRSNP/miRSNP_detail_all.php) | -0.127 |
| 39804218 | [hsa-miR-1292-3p](http://microrna.sanger.ac.uk/cgi-bin/sequences/mirna_entry.pl?acc=) | [rs73576045](http://www.ncbi.nlm.nih.gov/SNP/snp_ref.cgi?rs=rs73576045) | [C/T]GCGCCC | C/T | 0 | GGGCGCAA | [3](http://compbio.uthsc.edu/miRSNP/miRSNP_detail_all.php) | -0.247 |
| 39804190 | [hsa-miR-4290](http://microrna.sanger.ac.uk/cgi-bin/sequences/mirna_entry.pl?acc=) | [rs182483446](http://www.ncbi.nlm.nih.gov/SNP/snp_ref.cgi?rs=rs182483446) | G[C/T]CCUCC | C/T | 0 | GAGGACA | [4](http://compbio.uthsc.edu/miRSNP/miRSNP_detail_all.php) | -0.179 |
| 39803530 | [hsa-miR-6791-3p](http://microrna.sanger.ac.uk/cgi-bin/sequences/mirna_entry.pl?acc=) | [rs200895660](http://www.ncbi.nlm.nih.gov/SNP/snp_ref.cgi?rs=rs200895660) | [G/T]CCUCCU | G/T | 0 | AGGAGGA | [4](http://compbio.uthsc.edu/miRSNP/miRSNP_detail_all.php) | -0.221 |
| 39803723 | [hsa-miR-6729-5p](http://microrna.sanger.ac.uk/cgi-bin/sequences/mirna_entry.pl?acc=) | [rs75036690](http://www.ncbi.nlm.nih.gov/SNP/snp_ref.cgi?rs=rs75036690) | GGGC[G/A]AG | G/A | 1 | UUGCCCA | [9](http://compbio.uthsc.edu/miRSNP/miRSNP_detail_all.php) | -0.066 |
| 39803529 | [hsa-miR-6891-3p](http://microrna.sanger.ac.uk/cgi-bin/sequences/mirna_entry.pl?acc=) | [rs2276448](http://www.ncbi.nlm.nih.gov/SNP/snp_ref.cgi?rs=rs2276448) | CCUC[A/C]UC | A/C | 0 | GAGGAGGA | [4](http://compbio.uthsc.edu/miRSNP/miRSNP_detail_all.php) | -1.215 |
| 39803399 | [hsa-miR-5090](http://microrna.sanger.ac.uk/cgi-bin/sequences/mirna_entry.pl?acc=) | [rs3823658](http://www.ncbi.nlm.nih.gov/SNP/snp_ref.cgi?rs=rs3823658) | C[G/A]GGGCA | G/A | 1 | GCCCUGA | [11](http://compbio.uthsc.edu/miRSNP/miRSNP_detail_all.php) | -0.241 |
| 39803538 | [hsa-miR-4749-3p](http://microrna.sanger.ac.uk/cgi-bin/sequences/mirna_entry.pl?acc=) | [rs148982635](http://www.ncbi.nlm.nih.gov/SNP/snp_ref.cgi?rs=rs148982635) | [G/A]CCCCUC | G/A | 1 | GAGGAGC | [5](http://compbio.uthsc.edu/miRSNP/miRSNP_detail_all.php) | -0.088 |
| 39803538 | [hsa-miR-4749-3p](http://microrna.sanger.ac.uk/cgi-bin/sequences/mirna_entry.pl?acc=) | [rs200056596](http://www.ncbi.nlm.nih.gov/SNP/snp_ref.cgi?rs=rs200056596) | GC[C/T]CCUC | C/T | 0 | GAGGAGC | [5](http://compbio.uthsc.edu/miRSNP/miRSNP_detail_all.php) | -0.088 |
| 39803336 | [hsa-miR-1268a](http://microrna.sanger.ac.uk/cgi-bin/sequences/mirna_entry.pl?acc=) | [rs28599926](http://www.ncbi.nlm.nih.gov/SNP/snp_ref.cgi?rs=rs28599926) | GGGC[G/A]UG | G/A | 1 | CAUGCCC | [3](http://compbio.uthsc.edu/miRSNP/miRSNP_detail_all.php) | -0.162 |
| 39803332 | [hsa-miR-7847-3p](http://microrna.sanger.ac.uk/cgi-bin/sequences/mirna_entry.pl?acc=) | [rs148042410](http://www.ncbi.nlm.nih.gov/SNP/snp_ref.cgi?rs=rs148042410) | [G/A]UGGAGG | G/A | 1 | CCUCCAU | [5](http://compbio.uthsc.edu/miRSNP/miRSNP_detail_all.php) | -0.108 |
| 39804146 | [hsa-miR-6763-3p](http://microrna.sanger.ac.uk/cgi-bin/sequences/mirna_entry.pl?acc=) | [rs3751304](http://www.ncbi.nlm.nih.gov/SNP/snp_ref.cgi?rs=rs3751304) | UCCC[C/T]GG | C/T | 0 | CCAGGGA | [2](http://compbio.uthsc.edu/miRSNP/miRSNP_detail_all.php) | -0.37 |
| 39804031 | [hsa-miR-1238-3p](http://microrna.sanger.ac.uk/cgi-bin/sequences/mirna_entry.pl?acc=) | [rs200136327](http://www.ncbi.nlm.nih.gov/SNP/snp_ref.cgi?rs=rs200136327) | UUCCUC[G/A] | G/A | 1 | UGAGGAA | [3](http://compbio.uthsc.edu/miRSNP/miRSNP_detail_all.php) | -0.077 |
| 39803495 | [hsa-miR-5007-5p](http://microrna.sanger.ac.uk/cgi-bin/sequences/mirna_entry.pl?acc=) | [rs191336981](http://www.ncbi.nlm.nih.gov/SNP/snp_ref.cgi?rs=rs191336981) | [A/G]GAGUCU | A/G | 1 | AGACUCCA | [2](http://compbio.uthsc.edu/miRSNP/miRSNP_detail_all.php) | -0.252 |
| 39804104 | [hsa-miR-6736-3p](http://microrna.sanger.ac.uk/cgi-bin/sequences/mirna_entry.pl?acc=) | [rs139309317](http://www.ncbi.nlm.nih.gov/SNP/snp_ref.cgi?rs=rs139309317) | CAGCU[C/T]C | C/T | 0 | GAAGCUG | [7](http://compbio.uthsc.edu/miRSNP/miRSNP_detail_all.php) | -0.163 |
| 39803668 | [hsa-miR-1973](http://microrna.sanger.ac.uk/cgi-bin/sequences/mirna_entry.pl?acc=) | [rs183399490](http://www.ncbi.nlm.nih.gov/SNP/snp_ref.cgi?rs=rs183399490) | CC[G/A]UGCA | G/A | 1 | UGCAUGGA | [2](http://compbio.uthsc.edu/miRSNP/miRSNP_detail_all.php) | -0.174 |
| 39804144 | [hsa-miR-6742-3p](http://microrna.sanger.ac.uk/cgi-bin/sequences/mirna_entry.pl?acc=) | [rs138408187](http://www.ncbi.nlm.nih.gov/SNP/snp_ref.cgi?rs=rs138408187) | CCUGG[G/A]U | G/A | 1 | AUCCAGG | [2](http://compbio.uthsc.edu/miRSNP/miRSNP_detail_all.php) | -0.167 |
| 39804040 | [hsa-miR-1248](http://microrna.sanger.ac.uk/cgi-bin/sequences/mirna_entry.pl?acc=) | [rs202149708](http://www.ncbi.nlm.nih.gov/SNP/snp_ref.cgi?rs=rs202149708) | CCUU[C/T]UU | C/T | 0 | AAAAAGG | [3](http://compbio.uthsc.edu/miRSNP/miRSNP_detail_all.php) | -0.103 |
| 39803721 | [hsa-miR-6717-5p](http://microrna.sanger.ac.uk/cgi-bin/sequences/mirna_entry.pl?acc=) | [rs150596480](http://www.ncbi.nlm.nih.gov/SNP/snp_ref.cgi?rs=rs150596480) | GGC[G/A]AUG | G/A | 1 | CAUUGCC | [9](http://compbio.uthsc.edu/miRSNP/miRSNP_detail_all.php) | -0.093 |
| 39803721 | [hsa-miR-6717-5p](http://microrna.sanger.ac.uk/cgi-bin/sequences/mirna_entry.pl?acc=) | [rs117650137](http://www.ncbi.nlm.nih.gov/SNP/snp_ref.cgi?rs=rs117650137) | GG[C/T]GAUG | C/T | 0 | CAUUGCC | [9](http://compbio.uthsc.edu/miRSNP/miRSNP_detail_all.php) | -0.093 |
| 39803585 | [hsa-miR-4477b](http://microrna.sanger.ac.uk/cgi-bin/sequences/mirna_entry.pl?acc=) | [rs143791185](http://www.ncbi.nlm.nih.gov/SNP/snp_ref.cgi?rs=rs143791185) | UUAA[G/A]GA | G/A | 1 | CUUUAAA | [16](http://compbio.uthsc.edu/miRSNP/miRSNP_detail_all.php) | 0.068 |
