# Supplementary material for: A computational in silico approach to predict high-risk coding and non-coding SNPs of human PLCG1 gene
Source: PLoS One. 2021 Nov 18;16(11):e0260054. doi: 10.1371/journal.pone.0260054 (PMC8601573; doi:10.1371/journal.pone.0260054)
Supplement: S7 Table — (DOCX) [file pone.0260054.s007.docx]

**S1 Table 7. Regulome DB results**

| **chromosome** | **start** | **end** | **rsids** | **Probability** | **Ranking** | **ChIP** | **DNase** | **Footprint** | **Footprint**  **matched** | **IC_matched_max** | **IC_max** | **PWM** | **PWM_matched** | **QTL** |
| --- | --- | --- | --- | --- | --- | --- | --- | --- | --- | --- | --- | --- | --- | --- |
| chr20 | 39805246 | 39805247 | rs190546661 | 0.13454 | 5 | FALSE | TRUE | FALSE | FALSE | 0 | 0 | FALSE | FALSE | FALSE |
| chr20 | 39805098 | 39805099 | rs189708919 | 0.35267 | 5 | FALSE | TRUE | FALSE | FALSE | 0 | 0.41 | TRUE | FALSE | FALSE |
| chr20 | 39803715 | 39803716 | rs139043247 | 0.6 | 2a | TRUE | TRUE | TRUE | TRUE | 0.889999986 | 1 | TRUE | TRUE | FALSE |
| chr20 | 39766181 | 39766182 | rs577650473 | 0.60906 | 4 | TRUE | TRUE | FALSE | FALSE | 0 | 0 | FALSE | FALSE | FALSE |
| chr20 | 39805038 | 39805039 | rs541228792 | 0.13454 | 5 | FALSE | TRUE | FALSE | FALSE | 0 | 0 | FALSE | FALSE | FALSE |
| chr20 | 39806044 | 39806045 | rs575666863 | 0.60906 | 4 | TRUE | TRUE | FALSE | FALSE | 0 | 0 | FALSE | FALSE | FALSE |
| chr20 | 39804150 | 39804151 | rs567552943 | 0.60906 | 4 | TRUE | TRUE | FALSE | FALSE | 0 | 0 | FALSE | FALSE | FALSE |
| chr20 | 39805738 | 39805739 | rs532229042 | 0.29248 | 3a | TRUE | TRUE | FALSE | FALSE | 0 | 0.45 | TRUE | FALSE | FALSE |
| chr20 | 39804811 | 39804812 | rs148792723 | 0.13454 | 5 | FALSE | TRUE | FALSE | FALSE | 0 | 0 | FALSE | FALSE | FALSE |
| chr20 | 39805953 | 39805954 | rs571170027 | 0.30476 | 3a | TRUE | TRUE | FALSE | FALSE | 0 | 0.34 | TRUE | FALSE | FALSE |
| chr20 | 39803482 | 39803483 | rs141628590 | 0.60906 | 4 | TRUE | TRUE | FALSE | FALSE | 0 | 0 | FALSE | FALSE | FALSE |
| chr20 | 39805583 | 39805584 | rs561164565, rs1568765849 | 0.13454 | 5 | FALSE | TRUE | FALSE | FALSE | 0 | 0 | FALSE | FALSE | FALSE |
| chr20 | 39766154 | 39766155 | rs557864479 | 0.60906 | 4 | TRUE | TRUE | FALSE | FALSE | 0 | 0 | FALSE | FALSE | FALSE |
| chr20 | 39805978 | 39805979 | rs535979515 | 0.81114 | 3a | TRUE | TRUE | FALSE | FALSE | 0 | 0.26 | TRUE | FALSE | FALSE |
| chr20 | 39803181 | 39803182 | rs62621919 | 0.72923 | 3a | TRUE | TRUE | FALSE | FALSE | 0 | 2 | TRUE | FALSE | FALSE |
| chr20 | 39766011 | 39766012 | rs568877348 | 0.60906 | 4 | TRUE | TRUE | FALSE | FALSE | 0 | 0 | FALSE | FALSE | FALSE |
| chr20 | 39804767 | 39804768 | rs557418080 | 0.13454 | 5 | FALSE | TRUE | FALSE | FALSE | 0 | 0 | FALSE | FALSE | FALSE |
| chr20 | 39766201 | 39766202 | rs540297098 | 0.60906 | 4 | TRUE | TRUE | FALSE | FALSE | 0 | 0 | FALSE | FALSE | FALSE |
| chr20 | 39805775 | 39805776 | rs528215524 | 0.60906 | 4 | TRUE | TRUE | FALSE | FALSE | 0 | 0 | FALSE | FALSE | FALSE |
| chr20 | 39805200 | 39805201 | rs531996753 | 0.3882 | 5 | FALSE | TRUE | FALSE | FALSE | 0 | 1.79 | TRUE | FALSE | FALSE |
| chr20 | 39806107 | 39806108 | rs543218136 | 0.60906 | 4 | TRUE | TRUE | FALSE | FALSE | 0 | 0 | FALSE | FALSE | FALSE |
| chr20 | 39806029 | 39806030 | rs555596998 | 0.60906 | 4 | TRUE | TRUE | FALSE | FALSE | 0 | 0 | FALSE | FALSE | FALSE |
| chr20 | 39804237 | 39804238 | rs150066829 | 0.60906 | 4 | TRUE | TRUE | FALSE | FALSE | 0 | 0 | FALSE | FALSE | FALSE |
| chr20 | 39805972 | 39805973 | rs553378256 | 0.60906 | 4 | TRUE | TRUE | FALSE | FALSE | 0 | 0 | FALSE | FALSE | FALSE |
| chr20 | 39805812 | 39805813 | rs547958568 | 0.60906 | 4 | TRUE | TRUE | FALSE | FALSE | 0 | 0 | FALSE | FALSE | FALSE |
| chr20 | 39805254 | 39805255 | rs181874243 | 0.13454 | 5 | FALSE | TRUE | FALSE | FALSE | 0 | 0 | FALSE | FALSE | FALSE |
| chr20 | 39805714 | 39805715 | rs182769107 | 0.6352 | 3a | TRUE | TRUE | FALSE | FALSE | 0 | 1.93 | TRUE | FALSE | FALSE |
| chr20 | 39803769 | 39803770 | rs543804707 | 0.604 | 2b | TRUE | TRUE | TRUE | FALSE | 0 | 0.55 | TRUE | FALSE | FALSE |
| chr20 | 39805756 | 39805757 | rs559078953 | 0.60906 | 4 | TRUE | TRUE | FALSE | FALSE | 0 | 0 | FALSE | FALSE | FALSE |
| chr20 | 39805482 | 39805483 | rs182901101 | 0.13454 | 5 | FALSE | TRUE | FALSE | FALSE | 0 | 0 | FALSE | FALSE | FALSE |
| chr20 | 39805419 | 39805420 | rs537774643 | 0.13454 | 5 | FALSE | TRUE | FALSE | FALSE | 0 | 0 | FALSE | FALSE | FALSE |
| chr20 | 39803341 | 39803342 | rs184484019 | 0.60906 | 4 | TRUE | TRUE | FALSE | FALSE | 0 | 0 | FALSE | FALSE | FALSE |
| chr20 | 39804701 | 39804702 | rs543753711 | 0.13454 | 5 | FALSE | TRUE | FALSE | FALSE | 0 | 0 | FALSE | FALSE | FALSE |
| chr20 | 39803202 | 39803203 | rs375923042 | 0.60906 | 4 | TRUE | TRUE | FALSE | FALSE | 0 | 0 | FALSE | FALSE | FALSE |
| chr20 | 39804431 | 39804432 | rs558215724 | 0.60906 | 4 | TRUE | TRUE | FALSE | FALSE | 0 | 0 | FALSE | FALSE | FALSE |
| chr20 | 39805603 | 39805604 | rs543180961 | 0.13454 | 5 | FALSE | TRUE | FALSE | FALSE | 0 | 0 | FALSE | FALSE | FALSE |
| chr20 | 39803521 | 39803522 | rs41283256 | 0.60906 | 4 | TRUE | TRUE | FALSE | FALSE | 0 | 0 | FALSE | FALSE | FALSE |
| chr20 | 39805409 | 39805410 | rs190713195 | 0.13454 | 5 | FALSE | TRUE | FALSE | FALSE | 0 | 0 | FALSE | FALSE | FALSE |
| chr20 | 39804149 | 39804150 | rs547246451 | 0.60906 | 4 | TRUE | TRUE | FALSE | FALSE | 0 | 0 | FALSE | FALSE | FALSE |
| chr20 | 39805266 | 39805267 | rs534383223 | 0.13454 | 5 | FALSE | TRUE | FALSE | FALSE | 0 | 0 | FALSE | FALSE | FALSE |
| chr20 | 39805975 | 39805976 | rs114288140 | 0.66203 | 3a | TRUE | TRUE | FALSE | FALSE | 0 | 0.05 | TRUE | FALSE | FALSE |
| chr20 | 39805531 | 39805532 | rs186372122 | 0.13454 | 5 | FALSE | TRUE | FALSE | FALSE | 0 | 0 | FALSE | FALSE | FALSE |
| chr20 | 39803603 | 39803604 | rs146184925 | 0.60906 | 4 | TRUE | TRUE | FALSE | FALSE | 0 | 0 | FALSE | FALSE | FALSE |
| chr20 | 39804777 | 39804778 | rs144371341 | 0.13454 | 5 | FALSE | TRUE | FALSE | FALSE | 0 | 0 | FALSE | FALSE | FALSE |
| chr20 | 39804693 | 39804694 | rs34325936 | 0.13454 | 5 | FALSE | TRUE | FALSE | FALSE | 0 | 0 | FALSE | FALSE | FALSE |
| chr20 | 39803828 | 39803829 | rs532698922 | 0.60906 | 4 | TRUE | TRUE | FALSE | FALSE | 0 | 0 | FALSE | FALSE | FALSE |
| chr20 | 39805132 | 39805133 | rs549644731 | 0.13454 | 5 | FALSE | TRUE | FALSE | FALSE | 0 | 0 | FALSE | FALSE | FALSE |
| chr20 | 39803867 | 39803868 | rs546294729 | 0.60906 | 4 | TRUE | TRUE | FALSE | FALSE | 0 | 0 | FALSE | FALSE | FALSE |
| chr20 | 39803790 | 39803791 | rs184072600 | 0.60906 | 4 | TRUE | TRUE | FALSE | FALSE | 0 | 0 | FALSE | FALSE | FALSE |
| chr20 | 39804865 | 39804866 | rs559907751 | 0.55411 | 5 | FALSE | TRUE | FALSE | FALSE | 0 | 1.99 | TRUE | FALSE | FALSE |
| chr20 | 39805750 | 39805751 | rs551768008 | 0.90505 | 3a | TRUE | TRUE | FALSE | FALSE | 0 | 1.96 | TRUE | FALSE | FALSE |
| chr20 | 39803563 | 39803564 | rs572284106 | 0.60906 | 4 | TRUE | TRUE | FALSE | FALSE | 0 | 0 | FALSE | FALSE | FALSE |
| chr20 | 39804696 | 39804697 | rs575088786 | 0.13454 | 5 | FALSE | TRUE | FALSE | FALSE | 0 | 0 | FALSE | FALSE | FALSE |
| chr20 | 39803338 | 39803339 | rs555837375 | 0.60906 | 4 | TRUE | TRUE | FALSE | FALSE | 0 | 0 | FALSE | FALSE | FALSE |
| chr20 | 39804412 | 39804413 | rs538373964 | 0.60906 | 4 | TRUE | TRUE | FALSE | FALSE | 0 | 0 | FALSE | FALSE | FALSE |
| chr20 | 39804548 | 39804549 | rs535246280 | 0.13454 | 5 | FALSE | TRUE | FALSE | FALSE | 0 | 0 | FALSE | FALSE | FALSE |
| chr20 | 39803164 | 39803165 | rs534106686 | 0.60906 | 4 | TRUE | TRUE | FALSE | FALSE | 0 | 0 | FALSE | FALSE | FALSE |
| chr20 | 39803228 | 39803229 | rs536267192 | 0.60906 | 4 | TRUE | TRUE | FALSE | FALSE | 0 | 0 | FALSE | FALSE | FALSE |
| chr20 | 39805547 | 39805548 | rs573327915 | 0.13454 | 5 | FALSE | TRUE | FALSE | FALSE | 0 | 0 | FALSE | FALSE | FALSE |
| chr20 | 39805602 | 39805603 | rs574532959 | 0.13454 | 5 | FALSE | TRUE | FALSE | FALSE | 0 | 0 | FALSE | FALSE | FALSE |
| chr20 | 39805532 | 39805533 | rs553362774 | 0.13454 | 5 | FALSE | TRUE | FALSE | FALSE | 0 | 0 | FALSE | FALSE | FALSE |
| chr20 | 39804045 | 39804046 | rs527496846 | 0.60906 | 4 | TRUE | TRUE | FALSE | FALSE | 0 | 0 | FALSE | FALSE | FALSE |
| chr20 | 39805331 | 39805332 | rs1555818945, rs186197363 | 0.0065 | 5 | FALSE | TRUE | FALSE | FALSE | 0 | 0.31 | TRUE | FALSE | FALSE |
| chr20 | 39804214 | 39804215 | rs549407717 | 0.60906 | 4 | TRUE | TRUE | FALSE | FALSE | 0 | 0 | FALSE | FALSE | FALSE |
| chr20 | 39803756 | 39803757 | rs575006215 | 0.60906 | 4 | TRUE | TRUE | FALSE | FALSE | 0 | 0 | FALSE | FALSE | FALSE |
