# Supplementary figures and images for: A computational in silico approach to predict high-risk coding and non-coding SNPs of human PLCG1 gene
Source: PLoS One. 2021 Nov 18;16(11):e0260054. doi: 10.1371/journal.pone.0260054 (PMC8601573; doi:10.1371/journal.pone.0260054)

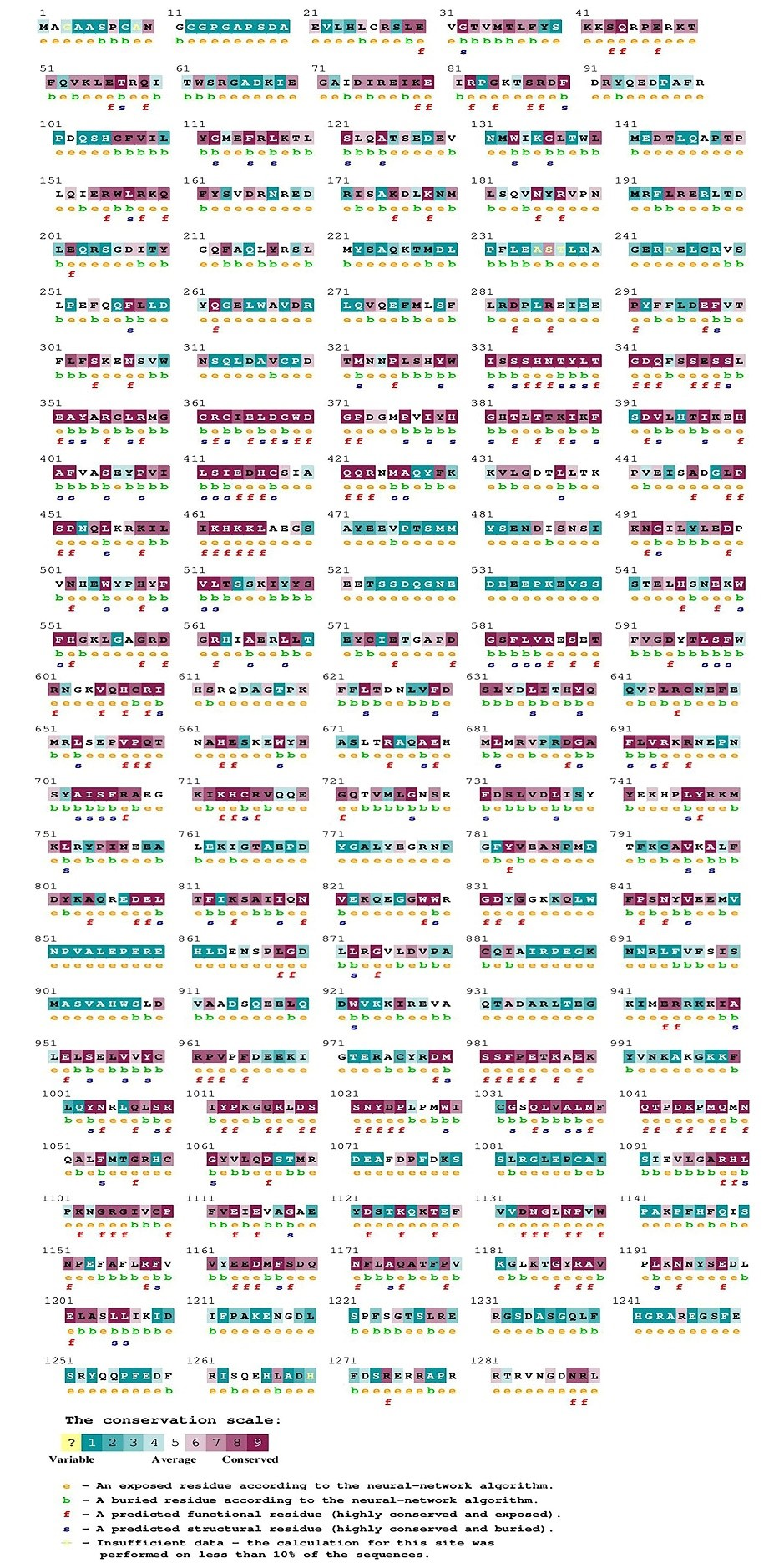

Supplement: S1 Fig — (TIF) [file pone.0260054.s008.tif]

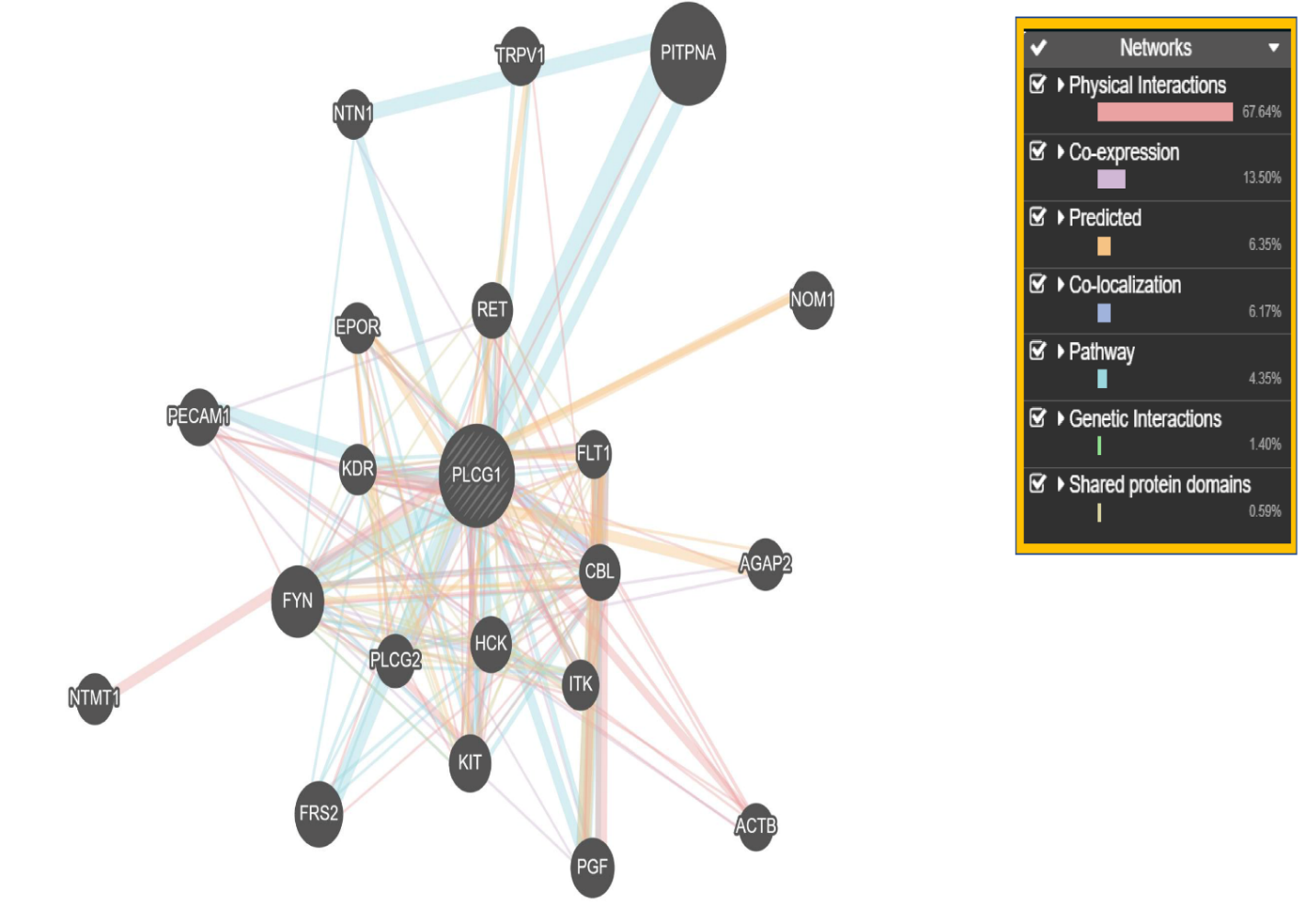

Supplement: S2 Fig — (TIF) [file pone.0260054.s009.tif]

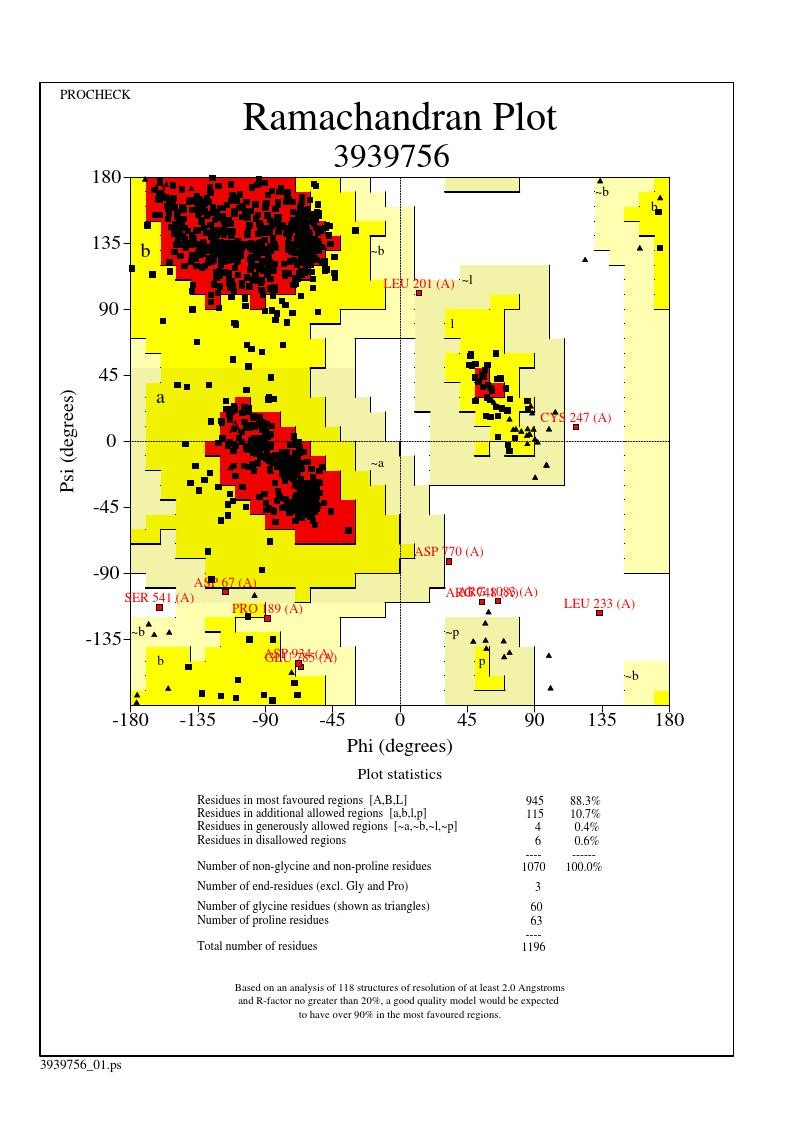

Supplement: S3 Fig — (TIF) [file pone.0260054.s010.tif]

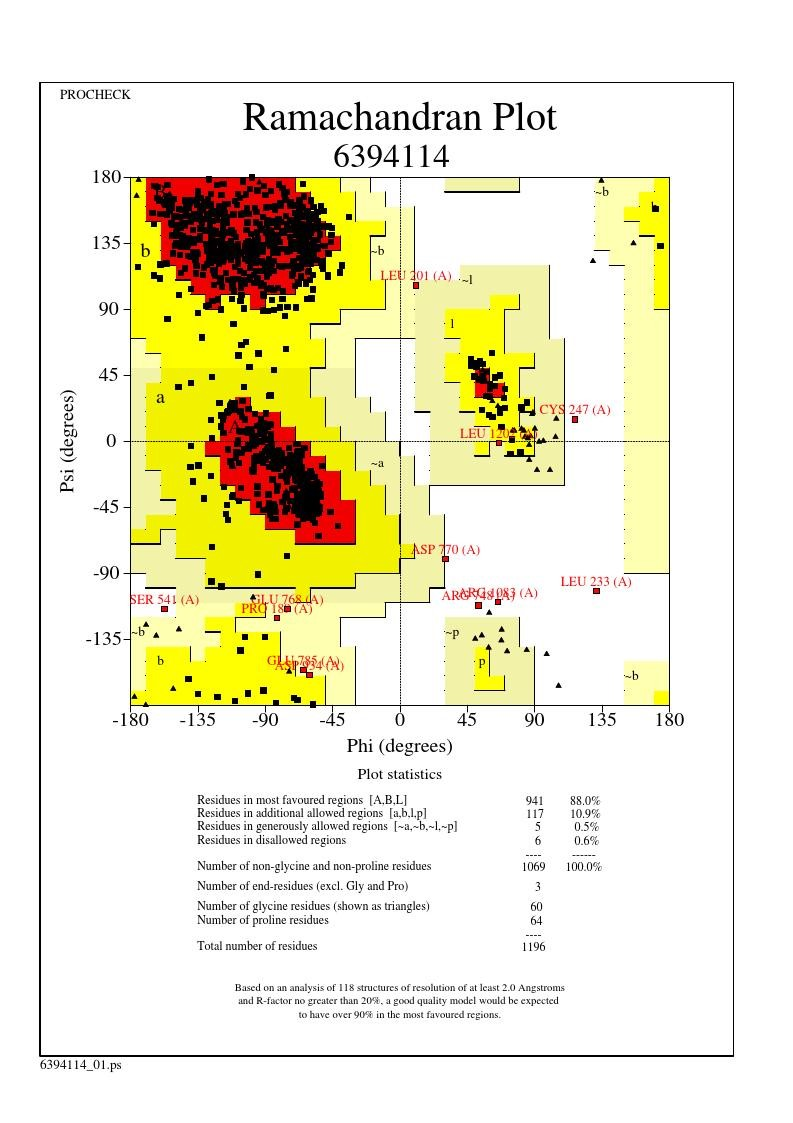

Supplement: S4 Fig — (TIF) [file pone.0260054.s011.tif]

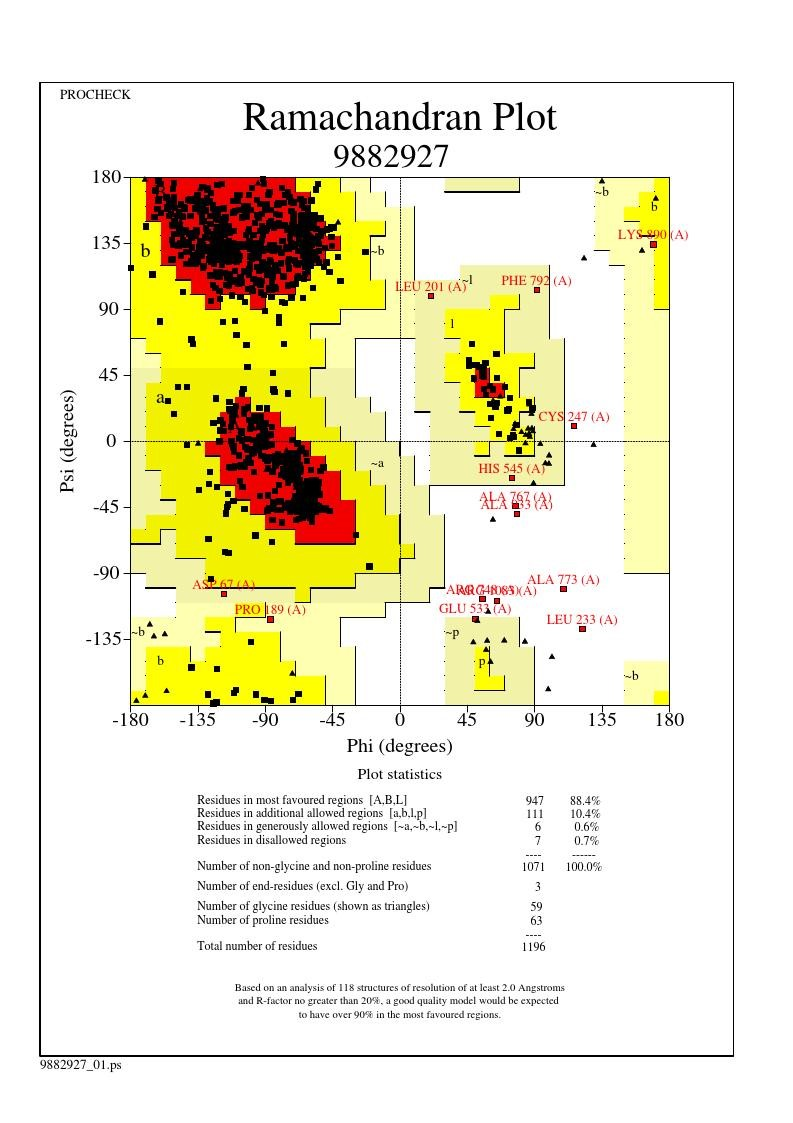

Supplement: S5 Fig — (TIF) [file pone.0260054.s012.tif]

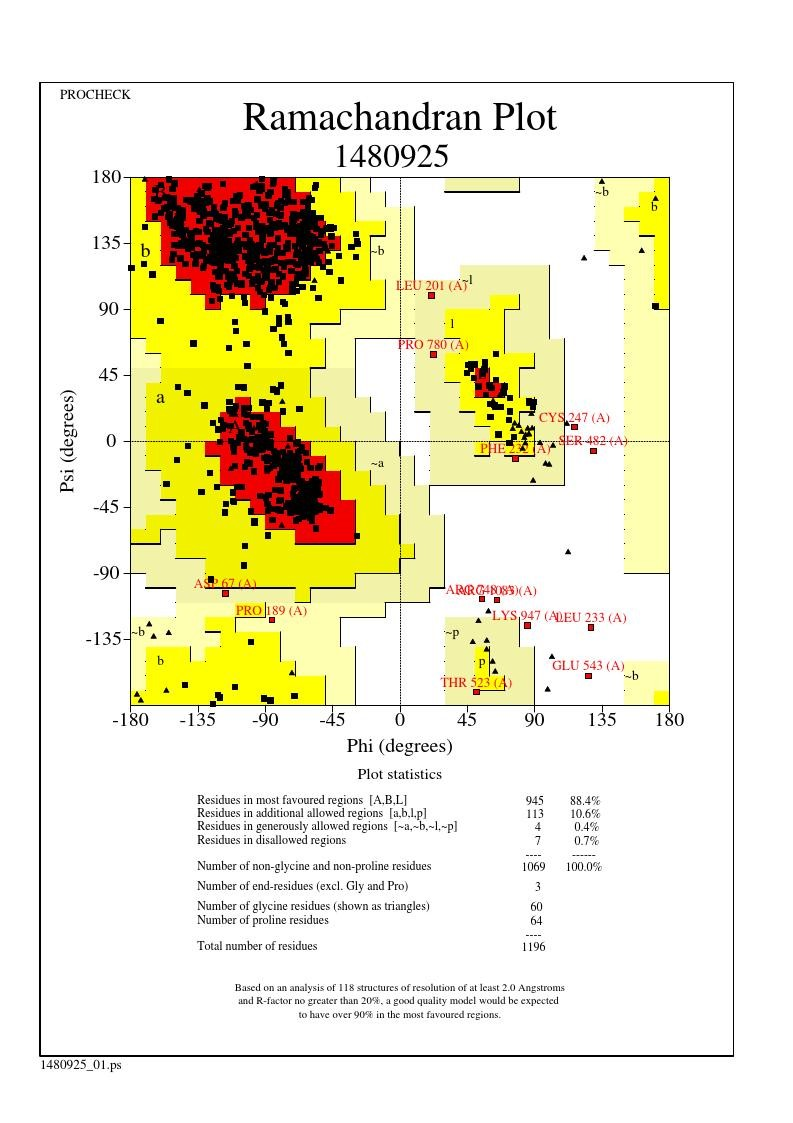

Supplement: S6 Fig — (TIF) [file pone.0260054.s013.tif]

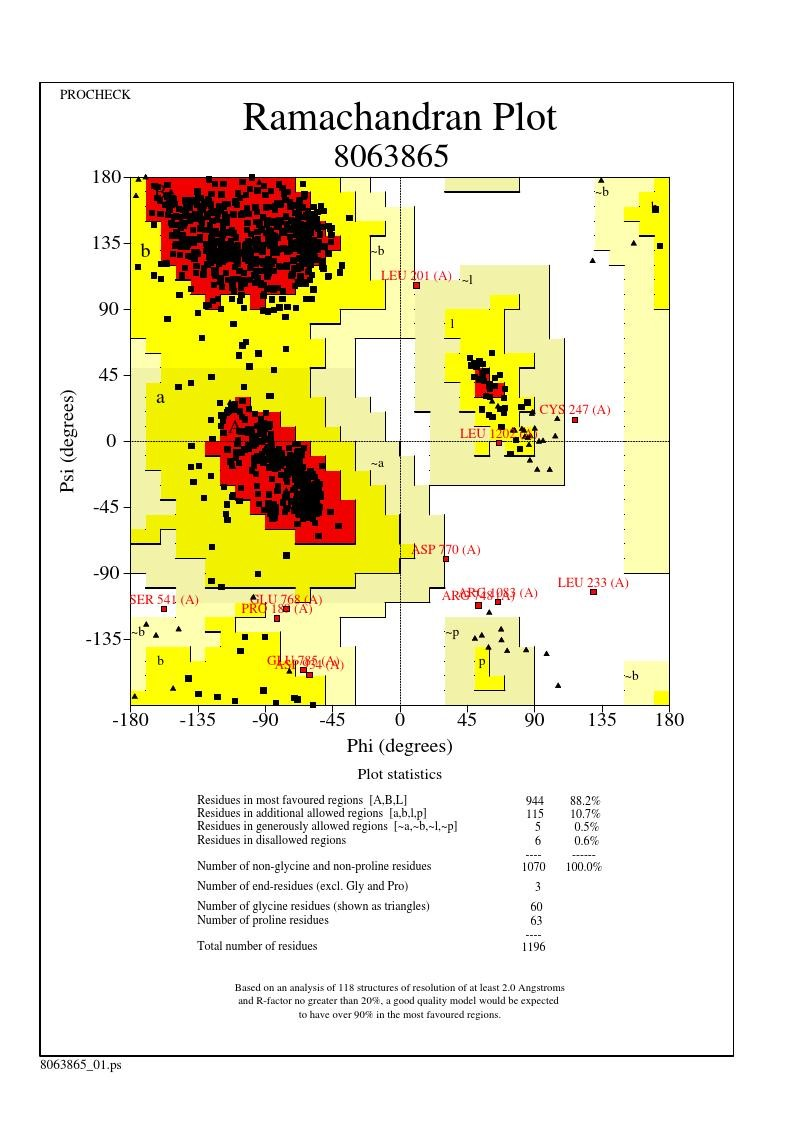

Supplement: S7 Fig — (TIF) [file pone.0260054.s014.tif]

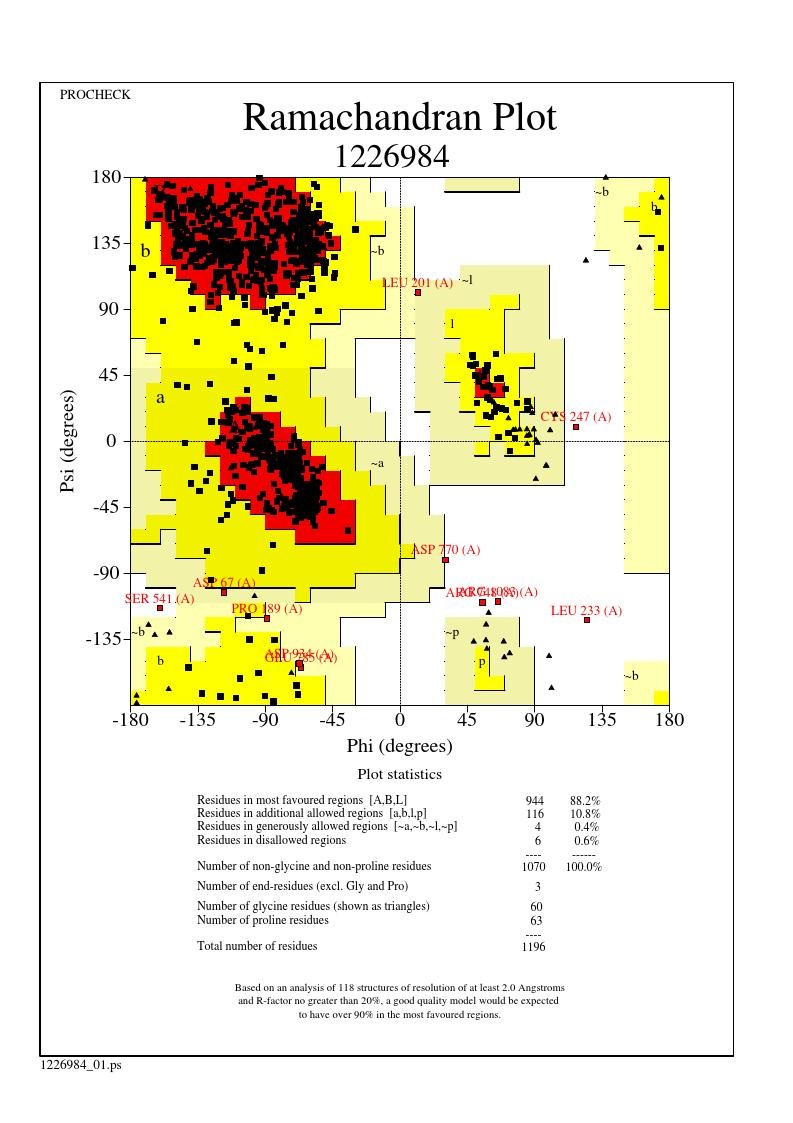

Supplement: S8 Fig — (TIF) [file pone.0260054.s015.tif]

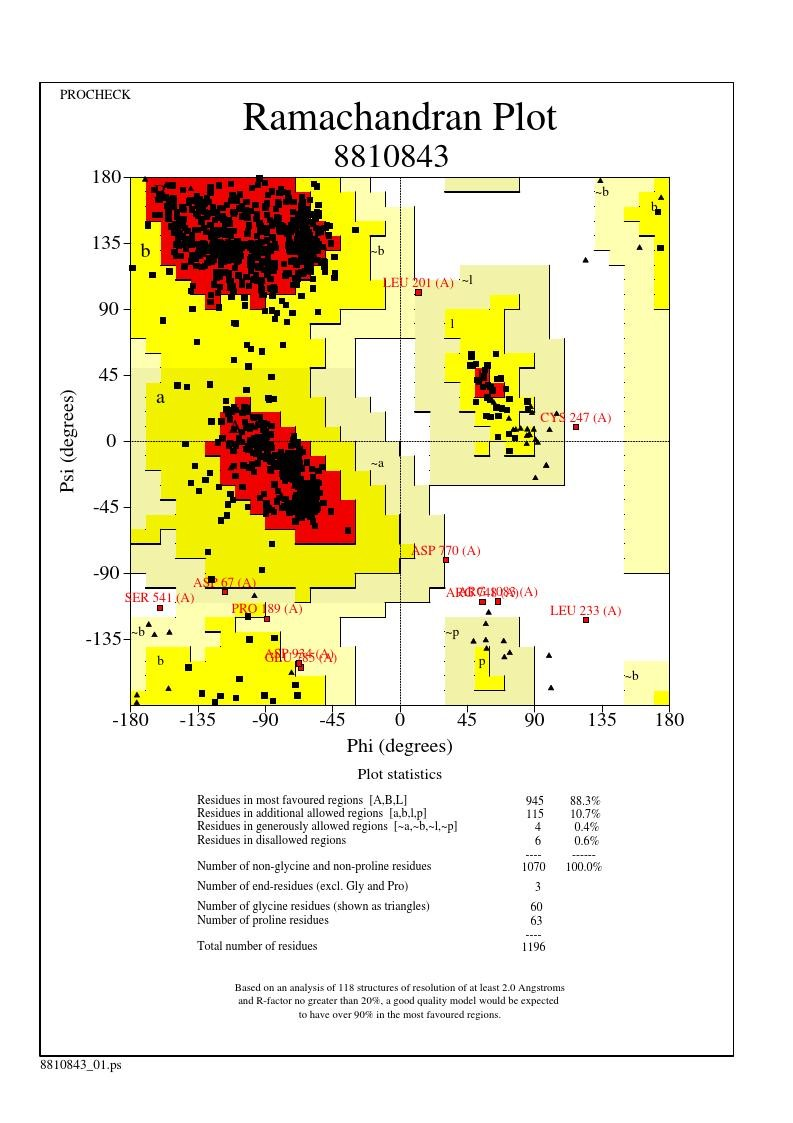

Supplement: S9 Fig — (TIF) [file pone.0260054.s016.tif]

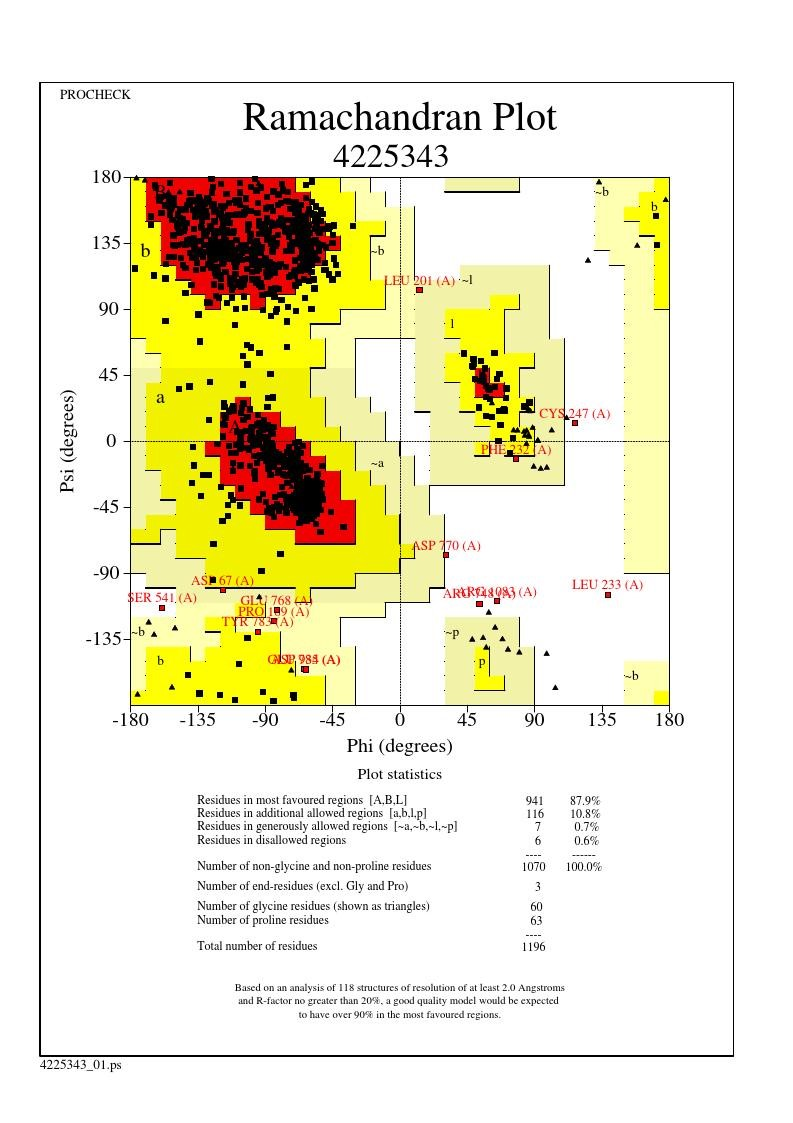

Supplement: S10 Fig — (TIF) [file pone.0260054.s017.tif]

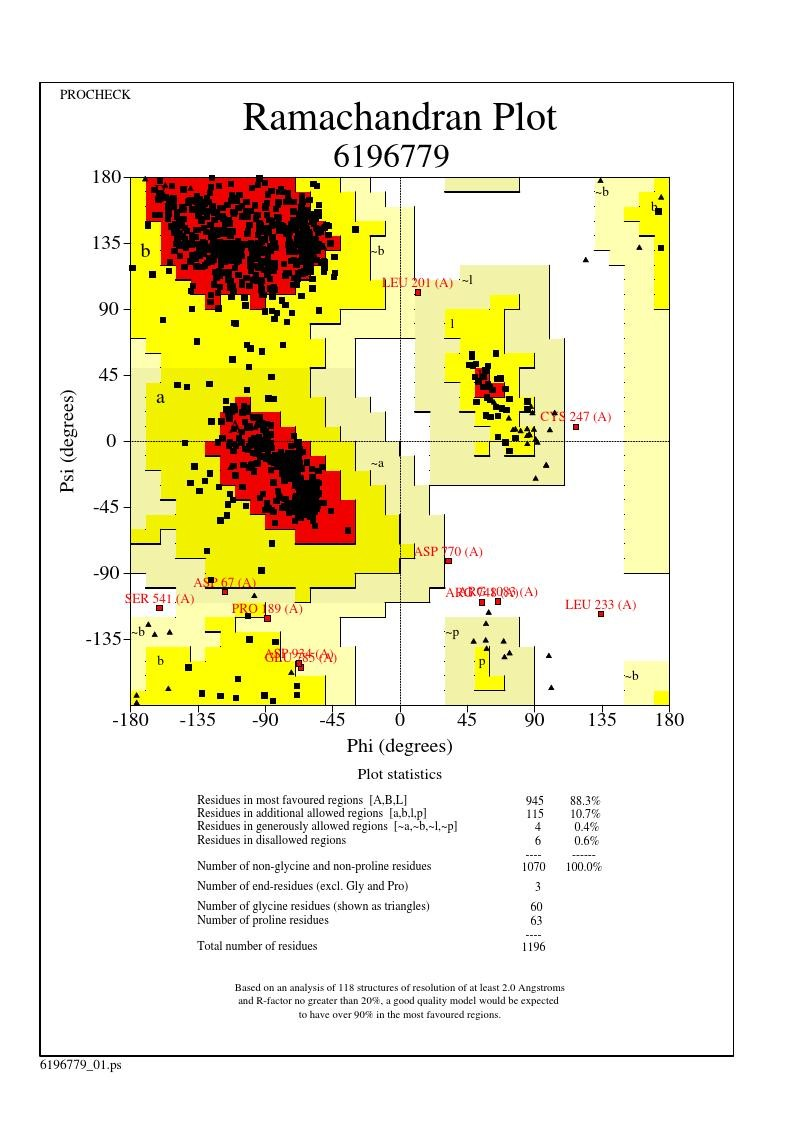

Supplement: S11 Fig — (TIF) [file pone.0260054.s018.tif]
